# The ANXA2P1-hnRNP F-HK2/c-Myc Positive Feedback Loop Promotes Proliferation and Glycolytic Metabolism in Gastric Cancer

**DOI:** 10.7150/ijbs.126842

**Published:** 2026-03-25

**Authors:** Ping Yang, Yanci Xie, Yuting Lei, Xiaodong Huang, Jieke Wu, Jiaxing Zhang, Siyang Peng, Yidong Chen, Xiangyang Wei, Jieming Zhang, Qiong Yang, Jiaying Li, Weiyu Dai, Xiaosheng Wu, Xinpeng Shi, Yanfeng Hu, Side Liu, Aimin Li, Weimei Tang, Jide Wang

**Affiliations:** 1 Guangdong Provincial Key Laboratory of Gastroenterology, Department of Gastroenterology, Nanfang Hospital, Southern Medical University, Guangzhou, 510515, China.; 2 Department of Gastroenterology, The Key Laboratory of Advanced Interdisciplinary Studies Center, The First Affiliated Hospital of Guangzhou Medical University, Guangzhou, 510120, China.; 3 Department of Gastroenterology and Hepatology, Guangdong Provincial People's Hospital, Guangdong Academy of Medical Sciences, Southern Medical University, Guangzhou, 510080, China.; 4 Department of Radiation Oncology, Luoyang Central Hospital Affiliated to Zhengzhou University, Luoyang, 471000, China.; 5 Department of General Surgery, Nanfang Hospital, Southern Medical University, Guangzhou, 510515, China.

**Keywords:** glycolysis, pseudogene, RNA metabolism, transcription

## Abstract

Pseudogene-derived long non-coding RNAs (lncRNAs) contribute to carcinogenesis. However, the role of the pseudogene ANXA2P1 in gastric cancer (GC) growth and glucose metabolism remains unknown. Analysis of microarray and RNA sequencing (RNA-seq) reveals that ANXA2P1 is increased upon glucose starvation in GC cells and displays elevated expression in GC. Moreover, ANXA2P1 overexpression promotes proliferation and metastasis by enhancing aerobic glycolysis in GC. Mechanistically, ANXA2P1 binds to the RNA-binding protein hnRNP F and promotes proximal polyadenylation site usage of HK2, thereby generating a short 3′UTR isoform with enhanced stability. Consequently, elevated HK2 expression accelerates GC proliferation and metabolic reprogramming. Interestingly, HK2 exerts a non-metabolic role by serving as a co-activator of transcription factor c-Myc to collaboratively drive ANXA2P1 expression. Clinically, ANXA2P1, hnRNP F, HK2, and c-Myc were augmented in specimens from GC patients compared to matched normal gastric mucosa. This study illustrates that ANXA2P1 is considered an oncogene, and the ANXA2P1-hnRNP F-HK2/c-Myc positive feedback loop may act as a potential therapeutic target for GC.

## Introduction

Gastric cancer (GC) is the 5^th^ most predominant malignancy and the 4^th^ key cause of cancer-correlated mortalities, with over one million new cases diagnosed yearly 1. Despite significant development in GC diagnosis and treatment over the previous decades, the prognosis of GC is still suboptimal worldwide 2. To develop more effective treatments, the molecular mechanisms of GC oncogenesis and development must be revealed.

Long noncoding RNAs (lncRNAs) are transcripts longer than 200 nucleotides that possess limited or no protein-coding potential. They significantly contribute to carcinogenesis by acting as either tumor suppressors or oncogenes 3, 4. Advances in deep sequencing have uncovered novel types of lncRNAs, including those derived from pseudogenes 5, 6. Usually, pseudogenes form by duplication or retrotransposition, and loss of gene function by disabling mutations. Recent investigations illustrated that pseudogenes participate in multiple cancer-related biological processes 6-8. For example, pseudogene DUXAP10 promotes cell growth and invasion by interacting with LSD1 in non-small cell lung cancer (NSCLC) 7. Pseudogene PIN1P1 triggers the GC tumorigenesis and metastasis by binding to YBX1 8. ANXA2P1 is a less well-investigated pseudogene-derived lncRNA positioned on chromosome 4q31.3 9. A previous study revealed that ANXA2P1, ANXA2P2, and ANXA2P3, accompanied by their parental gene annexin A2 (ANXA2, location: 15q22.2), were significantly upregulated in glioma 10. Moreover, pseudogene ANXA2P1 acts as a ceRNA to regulate the expression of its parental gene ANXA2 by serving as a sponge for miR-376a-3p in hepatocellular carcinoma (HCC) 9. Nonetheless, the expression and mechanisms of ANXA2P1 in GC are still unclear.

Aerobic glycolysis, also called the Warburg effect, has a vital function in metabolic reprogramming and tumor development 11-13. Increasing evidence indicates that many genes affect tumor cell survival via glycolysis under glucose deprivation conditions. For example, the LKB1-AMPK pathway promotes cell survival during glucose deprivation in cancer cells 12, and colorectal cancer (CRC) cells upregulate lncRNA GLCC1, which promotes cell survival and growth by enhancing glycolysis under glucose starvation 13. Recent progress has revealed that pseudogenes influence glucose metabolism in cancer. For instance, Nur77-stimulated pseudogene WFDC21P lessens hepatocarcinogenesis via regulating glycolysis 14. The pseudogene PDIA3P1 promotes ESCC progression by enhancing glycolysis 15. Nevertheless, the pseudogene ANXA2P1, which regulates aerobic glycolysis in GC cells, requires further investigation.

In this study, we concentrated on the roles of the pseudogene ANXA2P1 in the GC tumorigenesis and Warburg effect. Further experiments were designed to examine the ANXA2P1 biological function and its potential mechanism in GC progression. Herein, we showed an increase in ANXA2P1 expression and identified a novel signaling pathway, the ANXA2P1-hnRNP F-HK2 axis. Furthermore, we observed that ANXA2P1 was transcriptionally activated by c-Myc and co-factor HK2, thus forming the ANXA2P1-hnRNP F-HK2/c-Myc positive feedback loop, thereby contributing to the proliferation and glycolysis of GC.

## Materials and Methods

### Cell culture and chemicals

The Type Culture Collection of the Chinese Academy of Sciences (Shanghai, China) was the source of GC cell lines (AGS, MKN-45, SNU-216, SNU-1, SNU-5, HGC-27) and the normal gastric epithelial cell line GES-1, SNU-719 was purchased from Ubigene (China) and MKN-74 was purchased from Procell (China). Cells were cultured in Ham's F-12K, RPMI-1640, or DMEM (Gibco, USA) with 10% FBS (Biological Industries, Israel) at 37 °C in 5% CO₂. All cell lines were validated by STR-PCR and confirmed mycoplasma-free. For glucose starvation, glucose-free Ham's F-12K (Procell, China) or RPMI-1640 (Gibco, USA) was supplemented with D-glucose (Gibco, USA) to 1 mM. 2-NBDG was purchased from Invitrogen (USA), and 2-DG from Sigma (USA).

### RNA extraction and microarray analysis

Total RNA from AGS cells was extracted using TRIzol (Invitrogen, USA). RNA concentration and purity were assessed with a NanoDrop ND-2000 (Thermo Scientific, USA), and integrity was confirmed using an Agilent 2100 Bioanalyzer. Microarray analysis was performed by OEbiotech (China) following Agilent protocols. Briefly, RNA was reverse transcribed to cDNA, labeled cRNA was synthesized with Cyanine-3-CTP, hybridized to Agilent microarrays, and scanned using an Agilent G2505C Scanner. Feature Extraction software (v10.7.1.1, Agilent Technologies, USA) was utilized to extract raw data, and the quantile algorithm was utilized to normalize it. Probes detected in at least 75% of samples within any group were retained. Differentially expressed genes were identified based on thresholds set at |FC| > 1.2 and *P* value < 0.05. Tables S1 and S7 illustrate the glucose deprivation-associated lncRNAs and protein-coding RNAs. The lncRNA and mRNA microarray data have been deposited in the GEO under accession number GSE303312.

### Bioinformatics analysis

Differential expression analysis of pseudogenes was performed using the dreamBase database (https://rna.sysu.edu.cn/dreamBase2/). RNA-seq data (GSE122401), consisting of 79 paired GC and matched adjacent normal tissue samples, were downloaded from the NCBI Gene Expression Omnibus (GEO, http://www.ncbi.nlm.nih.gov/geo/). Expression levels of the candidate pseudogene ANXA2P1 across digestive system tumors and corresponding normal tissues were analyzed using the GEPIA platform (http://gepia.cancer-pku.cn/) with default settings. Survival data for TCGA stomach cancer (STAD) were acquired from the UCSC Xena TCGA hub (https://xenabrowser.net). Glycolysis-associated genes were retrieved from the KEGG database (https://www.genome.jp/kegg/). GO enrichment analysis was conducted via STRING (https://string-db.org/). RNA-protein interactions were predicted using the RPISeq tool (http://pridb.gdcb.iastate.edu/RPISeq). The interaction between ANXA2P1 and the HK2 3′UTR was predicted using IntaRNA, which was run locally with default parameters (http://rna.informatik.uni-freiburg.de/IntaRNA). Protein-protein interaction (PPI) data were obtained from the BioGRID database (https://thebiogrid.org/). Binding sites of transcription factor c-Myc in the promoter region of gene ANXA2P1 were predicted via JASPAR (https://jaspar.genereg.net/).

### Human GC tissue samples

Eighty GC specimens and matched adjacent normal tissues were collected from Nanfang Hospital, Southern Medical University (Guangzhou, China) between June 2017 and April 2020. All patients were treatment-naïve and diagnosed with primary GC by two pathologists. The investigation was conducted as per the Declaration of Helsinki of 1975 and received approval from the institutional ethics committee.

### RT-qPCR

cDNA synthesis was performed from 1 μg of total RNA via the Reverse Transcription Kit (TaKaRa, Japan). Quantitative PCR was conducted on a Roche LightCycler 480 system (Roche, Switzerland) via the SYBR Green PCR Kit (Takara, Japan). The 2^-ΔΔCt^ method was applied to measure the relative RNA levels between groups. Table S8A lists the Gene-specific primers.

### Cytoplasmic and nuclear RNA extraction

The Cytoplasmic and Nuclear RNA Purification Kit (Norgen Biotek Corporation, Canada) was utilized to isolate cytoplasmic and nuclear RNAs from 1 × 10^6^ GC cells as per the manufacturer's guidelines. ACTB and U6 served as endogenous controls for cytoplasmic and nuclear RNA, respectively.

### ISH

ISH was conducted to determine ANXA2P1 and HK2 levels in GC specimens using probe mixtures, each containing three nucleotide probes targeting distinct regions of ANXA2P1 or HK2. The probe sequences are as follows: ANXA2P1-probe-1: 5'-GTCTTGGTGCCCAGCCCAGCTTCCTTCAAAATATCTG-3'; ANXA2P1-probe-2: 5'-TGGCCACCTAGATAGTGGCTTTGGGCCTATTA-3'; ANXA2P1-probe-3: 5'-GCAAGACACTAAGGGTGCTGTACCTGTGTG-3'; HK2-probe-1: 5'-AGGATGGCATCCGGAAGGCCCGTGAGGTCCTGATGCGGTT-3'; HK2-probe-2: 5'-ACCCGACTCAGGAGGACTGCGTGGCCACTCACCGGATCTG-3'; HK2-probe-3: 5'-GCCAGAAGACATTAGAGCATCTGCAGCTGAGCCATGACCA-3'. Probes were synthesized and labeled with DIG (Boster, China). Hybridization signals were detected using an anti-DIG antibody followed by an alkaline phosphatase-conjugated secondary antibody, and visualized with NBT/BCIP (Sigma, USA) chromogenic substrate. Nuclear Fast Red was utilized to counterstain the sections prior to dehydration.

### H&E staining and IHC

The tissues that were embedded in paraffin were cut into 4 μm slices, exposed to xylene for deparaffinization, and then rehydrated using graded ethanol. The normal guidelines were followed to perform the H&E staining. In order to perform IHC, the antigen was heated in the sodium citrate buffer using a microwave. Before being incubated with 5% bovine serum albumin (BSA) for 30 min, slices were treated with 3% hydrogen peroxide to inhibit endogenous peroxidase. The primary antibodies were incubated at 4 °C for the night, followed by 1 h at room temperature with secondary antibodies conjugated with horseradish peroxidase (HRP). Staining was observed using 3,3′-diaminobenzidine (DAB) substrate (ZSGB-BIO, China).

### Western blot

Cell lysis was conducted in RIPA buffer with protease inhibitors (Sigma, USA), and protein concentration was measured using a BCA assay (Beyotime, China). Protein separation (25-50 μg/lane) was conducted via SDS-PAGE, transferred to PVDF membranes (Merck Millipore, USA), and an overnight incubation was conducted with primary antibodies at 4 °C, then with HRP-conjugated secondary antibodies for 1 h at room temperature. Protein bands were determined via an ECL reagent (Beyotime, China) and imaged using the Tanon 4800 system (Tanon, China). Table S8B lists the details of antibodies.

### Plasmids, shRNAs and dCas9/KRAB-sgRNAs

Plasmids including ANXA2P1, hnRNP F, HK2, c-Myc, truncated ANXA2P1 or hnRNP F, the wild-type or mutated promoter region of ANXA2P1, and full-length or mutant HK2 3′UTR were obtained from Kidan Biosciences (China). ShRNAs targeting hnRNP F, HK2, c-Myc, and IRF7 were received from GenePharma (China). Lentiviruses containing dCas9/KRAB and sgRNAs targeting ANXA2P1 were provided by Genechem Company (China). Table S8C lists the sequences.

### Cell growth, colony formation assays (CFA), and cell cycle analysis

Cell growth was measured via the CCK-8 (Yeasen, China) as per the manufacturer's guidelines. Cells (1 × 10³/well) were plated in 96-well plates, treated with CCK-8, then a 2-h incubation was conducted at 37 °C, and absorbance was recorded at 450 nm.

CFA was conducted by seeding 1 × 10³ cells/well in 12-well plates and culturing for 14 days. 4% paraformaldehyde was utilized to fix the colonies, and 0.5% crystal violet was utilized to stain them. Experiments were performed in triplicate.

A Cell Cycle Kit (Beyotime, China) was used for analyzing the cell cycle. The cells were fixed in 70% ethanol at 4 °C for 90 min, then stained with 0.5 mL of propidium iodide (PI) at 37 °C in the dark for 30 min, followed by analysis using the Beckman flow cytometer (Beckman Coulter, USA). The data was processed using the FlowJo program.

### Migration and invasion assays

Cell migration was evaluated via 24-well transwell chambers with 8 μm pore-size inserts (Corning, USA). GC cells (2 × 10⁴) were seeded in 200 μL serum-free medium in the top chamber, while 500 μL medium with 10% FBS was added to the bottom chamber as a chemoattractant. After 24 h of incubation, non-migrated cells were eliminated, and 4% paraformaldehyde was utilized to fix migrated cells, 0.5% crystal violet was utilized to stain them, and they were counted in five random fields per insert. For invasion assessments, Matrigel (BD Biosciences, USA) was utilized to pre-coat the inserts, and the same protocol was followed. All trials were conducted in triplicate.

### Wound healing assays

The GC cells were plated in 6-well plates and cultivated to near confluence. A 10 μL pipette tip was utilized to form a straight scratch across the middle of every well. After washing away detached cells, cultures were kept in 2% serum medium, and images were captured at specified time points to assess wound closure. The migration index was measured as: Migration index (%) = (initial wound width - wound width post-healing) / initial wound width × 100. Every trial was repeated at least three times.

### Glucose uptake and lactate production assessments

The GC cells (5 × 10^5^/well) were plated into 24-well plates and cultivated overnight. After adherence, a 12-h incubation of cells was conducted in serum-deprivation medium, followed by treatment with PBS for 40 min to induce metabolic stress. Then, 100 μL of full medium was applied to every well, and a 1-h incubation of cells was conducted at 37 °C. The culture supernatants were then collected, and glucose and lactate concentrations were determined via the Glucose Assay Kit (Abbkine, China) and the Lactate Assay Kit (Abbkine, China) as per the manufacturer's guidelines. All trials were carried out in triplicate.

### 2-NBDG uptake assay

The GC cells at 80%-90% confluence were rinsed with PBS and subjected to glucose-deprivation medium for 1 h, then an incubation was conducted with 50 μM 2-NBDG for an additional 1 h. After incubation, cell collection was conducted by a 5-min centrifugation at 1000 rpm, rinsing three times with PBS, and resuspending in 500 μL PBS. Glucose uptake was then assessed by flow cytometry via a CytoFLEX system (Beckman Coulter, USA).

### Measurement of extracellular acidification rate (ECAR)

ECAR was evaluated via the Seahorse XF96 Extracellular Flux Analyzer (Agilent Technologies, USA) following the manufacturer's guidelines. GC cells (1 × 10⁴ cells/well) were plated in XF96 plates and cultivated overnight. Before measurement, cells were incubated in unbuffered assay medium, and ECAR was recorded following sequential injection of 10 mM glucose, 1 μM oligomycin, and 50 mM 2-DG (2-deoxy-d-glucose). ECAR values were normalized to total cell number and expressed as mpH/min/10⁴ cells. All measurements were performed in triplicate.

### RNA pull-down and mass spectrometry (MS)

An RNA pull-down kit (BersinBio, China) was used to conduct the tests as per the guidelines of the manufacturer. RNAmax-T7 (RiboBio, China) was used to synthesize sense and antisense ANXA2P1 RNAs that were tagged with biotin *in vitro*. Cell lysates and streptavidin-conjugated magnetic beads were subjected to incubate with the biotinylated RNAs for 2 h at room temperature. After extensive washing, the RNA-protein complexes were eluted and analyzed by MS or Western blotting. MS was conducted by Fitgene Biotechnology (China), and Table S3A lists the proteins detected by MS.

### RNA-immunoprecipitation (RIP) and RIP-seq analysis

RIP assays were conducted using the Magna RIP RNA-Binding Protein Immunoprecipitation Kit (Merck Millipore, USA) following the manufacturer's guidelines. In brief, cell lysis was incubated with specific primary antibodies or control IgG conjugated to protein A/G magnetic beads. RNA was extracted from the immunoprecipitates, and enrichment was assessed by RT-qPCR. To identify the specific binding domain of hnRNP F, GC cell transfection was conducted with full-length or truncated FLAG-tagged hnRNP F plasmids. RIP was then performed using an anti-FLAG antibody. Table S8A-B illustrates the primer sequences and antibodies.

To profile hnRNP F-bound transcripts, RNA immunoprecipitates obtained using a hnRNP F-specific antibody were subjected to high-throughput sequencing. Library construction and sequencing were conducted by RiboBio (China) on the Illumina NovaSeq 6000 platform (Illumina, USA). The sequencing data have been submitted to the NCBI GEO database with accession number GSE303348, and Table S5 lists the hnRNP F-bound RNAs.

### mRNA-seq analysis following ANXA2P1 knockdown

Total RNA extraction was conducted from AGS cells transduced with CRISPRi-Scramble or CRISPRi-ANXA2P1 using TRIzol reagent (Invitrogen, USA), and RNA quality was evaluated with an Agilent 2100 Bioanalyzer (Agilent Technologies, USA). Library preparation and high-throughput sequencing were performed by Huayin Technology (China). Table S4 illustrates the differentially expressed protein-coding genes. The RNA-seq data have been submitted to the GEO with accession number GSE303469.

### RNA stability assay

The cells were placed onto 12-well plates and cultivated until approaching around 60% confluence. After that, they were exposed to 10 μg/mL actinomycin D (Sigma, USA) for the time points.

### RNA antisense purification (RAP)

A pool of 90-mer 5' biotin-labeled DNA probes specific for ANXA2P1 were synthesized by Ruibo (China), and assays were conducted using a RAP kit (BersinBio, China). Briefly, biotinylated probes pre-coupled to magnetic beads were incubated with the lysates. Following pull-down, the associated RNAs were bound to the streptavidin beads. After elution and purification, the captured RNAs were subsequently subjected to RT-qPCR.

### Chromatin immunoprecipitation (ChIP) and Re-ChIP analysis

ChIP assessments were performed via the SimpleChIP Enzymatic Chromatin IP Kit (Cell Signaling Technology, USA) as per the manufacturer's guidelines. In brief, GC cells were fixed with formaldehyde to cross-link protein-DNA interactions, followed by sonication to shear chromatin into fragments. Then, an overnight incubation of lysates was conducted at 4 °C with c-Myc antibody or control IgG. After extensive washing, the immunoprecipitated DNA was purified for downstream analysis.

To investigate the potential role of HK2 as a transcriptional cofactor, cells were subjected to transfection with His-tagged HK2, and ChIP assays targeting HK2 were performed using an anti-His antibody. For the Re-ChIP experiments, the first immunoprecipitation was conducted using an anti-c-Myc antibody. A 30-min elution of immune complexes was conducted with 10 mM DTT at 37 °C, followed by a 20-fold dilution and immunoprecipitation with an anti-His antibody. Subsequent procedures followed the same protocol as described above for the ChIP assay. Table S8A illustrates the primer sequences used for ChIP and Re-ChIP PCR.

### Coimmunoprecipitation (Co-IP) and DNA pull-down assay

Co-IP was conducted by an overnight incubation of cell lysates with a specific antibody at 4 °C, then capture with pre-cleared protein A/G agarose beads. After thorough washing, the immunoprecipitated complexes were assessed by Western blotting.

For DNA pull-down assessment, DNA probes (~2000 bp) were amplified by PCR using the same pGL3-basic-based plasmids containing full-length promoter sequences employed in the dual-luciferase reporter assays. For generating the biotin-labeled probe, 5′-biotinylated primers were used during PCR amplification. The DNA pull-down assay was performed using the DNA pull-down kit (BersinBio, China) according to the manufacturer's instructions. Briefly, protein lysates were incubated with DNA probes conjugated to streptavidin-agarose beads at 4 °C for 1 hour, followed by elution and Western blotting.

### Luciferase reporter assay for promoter activity

Cloning the full-length or mutant promoter sequences was performed by inserting them into the pGL3-basic vector. The mutant sequence at positions +110 to +117 was altered from 5′-CCAAGTGC-3′ to 5′-ACGAATAC-3′. GC cells were lysed with the Dual-Luciferase Reporter Assay Kit (Vazyme, China) as per the manufacturer's guidelines. To assess the activity of firefly and Renilla luciferase, the GloMax Luminometer (Promega, USA) was utilized. The ratio of firefly to Renilla luminescence was used to calculate the relative luciferase activity.

### Luciferase activity assessment for the 3′UTR analysis

Amplification and cloning of the full-length 3′UTR of HK2 were inserted downstream of the firefly luciferase coding area in the pmirGLO vector using NheI and SalI restriction sites. Mutant constructs were generated by replacing the "UGGGG" motif with "CGAGT" individually at four distinct sites within the 3′UTR. In addition, short and long 3′UTR reporters were constructed. The short 3′UTR reporter represents a truncated HK2 transcript ending at the proximal cleavage and polyadenylation site (chr2:74,891,162). For the long 3′UTR reporter, the proximal polyadenylation signal (pPAS) region within the HK2 3′UTR (chr2:74,891,126-74,891,147; containing the PAS motifs GATAAA, AATAGA, and AATAAG) was mutated to prevent usage of the pPAS and thereby enforce expression of the long 3′UTR isoform. Specifically, for each motif, nucleotides at positions 2 and 4 of the consensus were changed to cytosine (C). Dual-luciferase reporter assessments were conducted as described.

### 3′ Rapid Amplification of cDNA Ends (3′RACE)

To determine the 3′ end structure of HK2 transcripts, 3′RACE was performed using the 3′-Full RACE Core Set with PrimeScript RTase (Takara, Code No. 6106). Briefly, total RNA was isolated from cells and reverse-transcribed using PrimeScript reverse transcriptase and a 3′RACE adaptor primer that anneals to the poly(A) tail, generating first-strand cDNA. Nested PCR amplification was subsequently carried out using gene-specific outer and inner primers in combination with the corresponding 3′RACE outer and inner primers to specifically amplify the 3′ ends of HK2 transcripts. PCR products were resolved by agarose gel electrophoresis, purified, and subjected to sequencing. The obtained sequences were aligned to the human genome to identify cleavage and polyadenylation sites and to define distinct HK2 3′UTR isoforms. The sequences of gene-specific outer and inner primers are listed in Supplementary Table S8A.

### RNase H alternative polyadenylation assay (RHAPA)

RHAPA was performed as previously described to quantify alternative polyadenylation events 16, 17. Briefly, equal amounts of RNA were hybridized with a gene-specific antisense DNA oligonucleotide designed to anneal between the proximal and distal polyadenylation sites. RNA-DNA hybrids were then digested with RNase H to selectively cleave the RNA strand at the oligonucleotide hybridization site. After RNase H digestion, RNA was purified and reverse-transcribed with oligo(dT), thereby selectively generating cDNA from polyadenylated RNA fragments. RNase H cleavage was confirmed by PCR using primers spanning the oligonucleotide hybridization region, with products analyzed by agarose gel electrophoresis. Isoform-specific qRT-PCR was used to assess proximal and distal polyadenylation site usage.

### Generation and transduction of lentiviral vectors *in vitro*

Lentiviral constructs carrying antibiotic resistance markers (puromycin, geneticin, or blasticidin) and fluorescent reporters, were obtained from Obio Technology (China). The transduction of lentiviruses was performed on GC cells with 5 μg/mL polybrene. Stable cell lines were generated by antibiotic selection according to the resistance markers.

### Subcutaneous xenograft experiments

Guangdong Medical Laboratory Animal Center (China) supplied BALB/C (nu/nu, 4-6 weeks old) mice. To assess *in vivo* tumorigenesis, GC cells transduced with the indicated lentiviral constructs were resuspended, and 0.1 mL of cell suspension (5 × 10⁶ cells) was subcutaneously injected into the right flank of every mouse.

Three cohorts of mice from different groups were utilized to evaluate the proliferative capability of malignant cells. For the first batch of animal experiments, seven days after subcutaneous inoculation, mice were intraperitoneally administered 2-DG (500 mg kg^-1^; Sigma, USA) or vehicle control every other day for two weeks.

Tumor volumes were measured every three days via the formula: volume (mm³) = (length × width²)/2. After 21 days, mice were sacrificed under isoflurane anesthesia, then cervical dislocation. Tumors were harvested and subjected to H&E staining and IHC analysis. All animal trials received approval from the Experimental Animal Ethics Committee of Southern Medical University.

### Statistical analysis

SPSS v23.0 (SPSS Inc., USA), GraphPad Prism 8 (GraphPad Software, USA), and R v4.0.2 (R Foundation for Statistical Computing, Austria) were utilized to conduct statistical analyses. Data are reported as mean ± standard deviation (SD). Depending on study design and data distribution, appropriate tests were applied, including Student's t-test, Mann-Whitney U test, Wilcoxon signed-rank test, one-way ANOVA, Kruskal-Wallis test, Pearson correlation, and Kaplan-Meier survival analysis with log-rank test. The optimal cutoff value for high versus low expression of ANXA2P1 in survival analysis was determined using x-tile software (Yale University, USA). A two-tailed P value < 0.05 was recognized as significant. All *in vitro* trials were conducted in triplicate.

## Results

### ANXA2P1 is upregulated in GC

**Figure 1:**
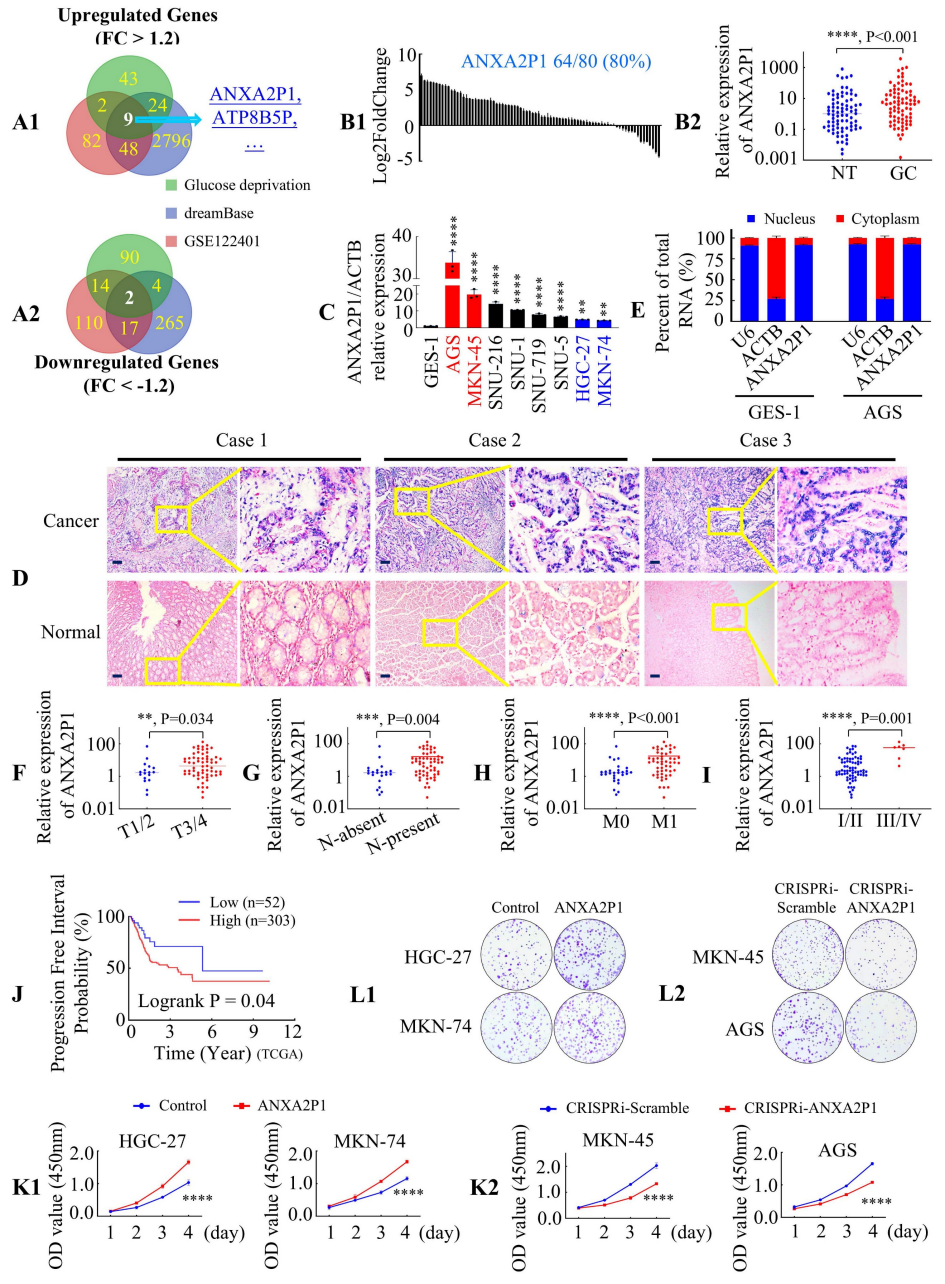
** ANXA2P1 is upregulated and correlated with development and progression in GC. (A1/2)** Venn diagram displays the differentially expressed pseudogenes between gastric cancer (GC) tissues and adjacent normal tissues from the GSE122401 dataset (https://www.ncbi.nlm.nih.gov/geo) and dreamBase database (https://rna.sysu.edu.cn/dreamBase/), along with those altered in AGS cells under glucose deprivation compared to normal-glucose medium conditions (threshold: |FC| ≥ 1.2). **(B1/2)** Relative ANXA2P1 expression levels in 80 paired GC and adjacent normal tissues (NT), measured by RT-qPCR, were presented as a waterfall plot (B1) and a scatter plot (B2). B2M was used as an internal control. GC, gastric cancer; NT, normal tissue. **(C)** ANXA2P1 expression in GC cell lines compared with normal gastric epithelial GES-1 cells. GC cell lines versus GES-1. **(D)** Representative *in situ* hybridization (ISH) images indicate the localization and expression of ANXA2P1 in GC and adjacent normal tissues. **(E)** Subcellular localization of ANXA2P1 in GES-1 and AGS cells. ACTB and U6 were used as controls for the cytoplasmic and nuclear fractions, respectively. **(F-I)** Dot plots indicate that ANXA2P1 expression was positively associated with local invasion (F), lymph-node metastasis (G), distant metastasis (H), and AJCC stage (I). **(J)** Kaplan-Meier curves for progression-free interval stratified by ANXA2P1 expression levels using the TCGA database (original source of dreamBase). **(K1/2)** CCK-8 proliferation assay was used to compare the proliferation rates of GC cells. **(L1/2)** Colony formation assay (CFA) was utilized to compare colony formation abilities. Scale bar: 100 µm in (D). Wilcoxon signed rank test (B2); one-way analysis of variance (ANOVA), Dunnett's multiple comparisons test (C); Mann-Whitney U test (F-I); Kaplan-Meier survival analysis with log-rank test (J); Student's t-test (K). **P < 0.05, ***P < 0.01, and ****P < 0.001. Data are presented as mean ± standard deviation (SD).

To investigate glycolysis-associated lncRNAs in GC, a microarray analysis was conducted to compare the lncRNA expression between low-glucose (LG) medium [1.0 mM] and normal-glucose (NG) medium [7.0 mM] in AGS cells 18. A total of 1402 differentially expressed lncRNAs were detected, involving 188 pseudogenes annotated by the HUGO Gene Nomenclature Committee (HGNC) (Table S1). Meanwhile, we downloaded lncRNA expression profiles from GSE122401 and pseudogene-specific data from dreamBase, both based on GC and adjacent normal tissues. The intersection of three high-throughput datasets identified nine upregulated and two downregulated pseudogene-derived lncRNAs (*P* < 0.05, |FC (Fold change) | > 1.2) (Figure 1A1-2). We then focused on the nine upregulated genes due to their potential as early diagnostic markers or therapeutic targets. Among them, eight had not been previously reported to be related to GC (based on the literature available up to August 1, 2024). To investigate these eight pseudogenes, we designed transcript-specific primers targeting unique sequences to distinguish them from their parental genes, and examined their levels in 40 pairs of matched GC and adjacent normal tissues using Real Time-qPCR (RT-qPCR). Compared to normal gastric tissues, the expression rates of RRN3P2, TMEM191A, CTAGE7P, HTATSF1P2, ZNF833P, ATP8B5P, OR7E47P, and ANXA2P1 in GC tissues were 45% (18/40), 50% (20/40), 50% (20/40), 52.5% (21/40), 55% (22/40), 55% (22/40), 60% (24/40), and 80% (32/40), respectively, with ANXA2P1 exhibiting the most pronounced upregulation (Figure S1A-H). These results were further confirmed using the dreamBase and GSE122401 databases using a scatter plot, which consistently showed significantly higher ANXA2P1 expression in GC tissues (Figure S1I-J). Moreover, the findings from the GEPIA database indicated that ANXA2P1 level was upregulated in a variety of digestive tract tumors, including GC, compared to adjacent normal tissues (Figure S1K).

To further validate our results, RT-qPCR analysis was expanded to 80 paired GC and adjacent normal tissues, demonstrating frequent upregulation of ANXA2P1 in GC tissues (Figure 1B1-2). Moreover, ANXA2P1 was overexpressed in all eight GC cell lines compared to the normal human gastric epithelial cell line GES-1 (Figure 1C). *In situ* hybridization (ISH) indicates that ANXA2P1 was mainly localized in GC tissue, while only weak signals were detected in normal tissues. Notably, strong ANXA2P1 signals were presented predominantly in the nucleus, while weaker signals in the cytoplasm of cancer cells (Figure 1D). Consistently, the cytoplasmic/nuclear fractionation assay also confirmed that ANXA2P1 showed a predominant localization in the nucleus, with partial cytoplasmic distribution in GES-1 and AGS cells (Figure 1E).

To explore the relationship between ANXA2P1 expression and clinical characteristics of GC, we performed statistical analyses, which revealed that ANXA2P1 expression was positively correlated with local invasion, lymph-node metastasis, distant metastasis, and AJCC stage (Figure 1F-I). ANXA2P1 levels are also linked to increased tumor size and poorer differentiation status, but show no significant association with patient gender or age (Table S2A). We examined ANXA2P1 expression and survival data from the TCGA database (the original source of dreamBase). Based on the appropriate threshold value calculated by x-tile, individuals were classified into low and high expression groups. Shorter progression-free interval (P = 0.04) and disease-specific survival (P = 0.038) were related to high ANXA2P1 expression, according to Kaplan-Meier analysis (Figure 1J and S1L). Although not statistically significant, high ANXA2P1 expression tended to be associated with poorer overall survival (Figure S1M) (P = 0.13). These outcomes illustrate that ANXA2P1 may trigger the development and progression of GC.

### ANXA2P1 promotes the growth and metastasis of GC

To further investigate the biological function of ANXA2P1 in GC cells, stable ANXA2P1-overexpressing (HGC-27 and MKN-74, with low endogenous expression) cell lines were established via lentiviral transduction. CRISPR interference (CRISPRi) was used to knock down ANXA2P1 expression (MKN-45 and AGS, with high endogenous expression), by introducing dCas9-KRAB and sequence-specific single-guide RNAs (sgRNAs) into cells. The efficiency was verified by RT-qPCR (Figure S2A). We then tested the effect of ANXA2P1 on growth and metastasis in GC cells. The outcomes illustrated that the forced expression of ANXA2P1 significantly triggered cell growth, as measured by Cell Counting Kit-8 (CCK-8) and colony formation assays (CFA). However, knockdown of ANXA2P1 suppressed cell proliferation in MKN-45 and AGS cells. (Figure 1K-L and S2B). We then examined ANXA2P1's role in cell cycle progression and its knockdown arrested cells in the G0/G1 phase using flow cytometry analysis (Figure S2C1-2), which was accompanied by Western blot results showing downregulation of CDK4/6 and Cyclin D1, while Cyclin B1 remained unchanged (Figure S2D).

Moreover, transwell assays with or without Matrigel demonstrated that the forced expression of ANXA2P1 in HGC-27 and MKN-74 cells enhanced the ability to migrate and invade, while ANXA2P1 knockdown in MKN-45 and AGS cells reduced these abilities (Figure S3A-B). Similar results were obtained in wound healing assays, indicating that elevated ANXA2P1 expression facilitated migration, while knockdown impeded it (Figure S3C-D). All these data suggest that ANXA2P1 promotes malignant phenotypes in GC cells.

### ANXA2P1 facilitates aerobic glycolysis of GC cells *in vitro* and *in vivo*

**Figure 2:**
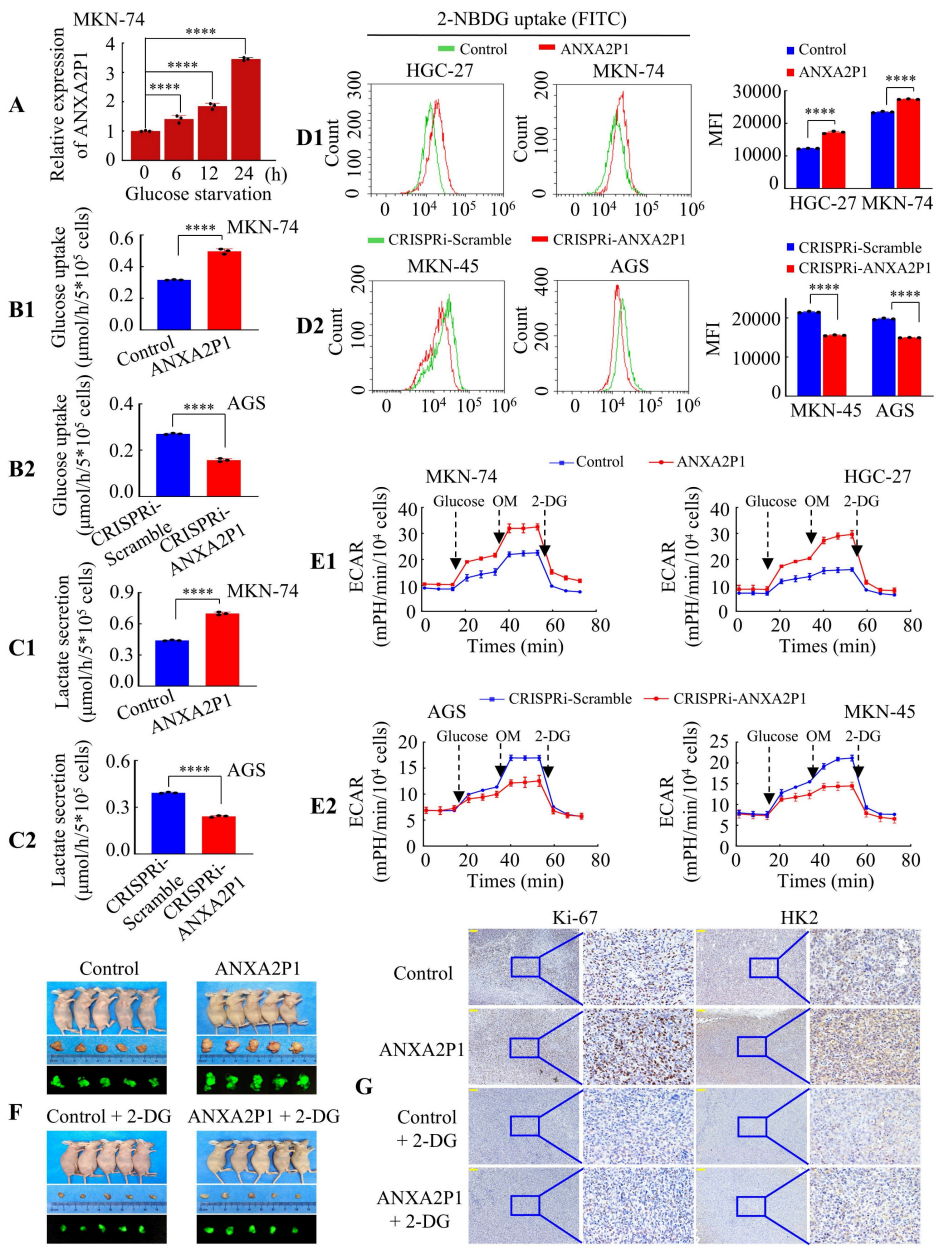
**ANXA2P1 promotes the glycolysis of GC cells**.** (A)** RT-qPCR analysis of ANXA2P1 expression in MKN-74 cells after incubation in low-glucose medium for 0, 6, 12, and 24 h. **(B1/2-C1/2)** Glucose consumption (B) and lactate level (C) were measured in the supernatants of MKN-74 and AGS cells after treated with control or ANXA2P1 plasmid (B1 and C1), or with CRISPRi-Scramble or CRISPRi-ANXA2P1 (B2 and C2). **(D1/2)** Flow cytometry analysis of 2-NBDG uptake in GC cells revealed that ANXA2P1 overexpression significantly increased (D1), while ANXA2P1 knockdown notably decreased (D2) glucose uptake. 2-NBDG, fluorescent d-glucose analog 2-[N-(7-nitrobenz-2-oxa-1,3-diazol-4-yl) amino]-2-deoxy-D-glucose. **(E1/2)** Extracellular acidification rate (ECAR) was measured in GC cells after transfection with control or ANXA2P1 (E1), or with CRISPRi-Scramble or CRISPRi-ANXA2P1 (E2). OM, oligomycin; 2-DG, 2-deoxyglucose. **(F)** Representative images of subcutaneous tumors of mice injected with MKN-74 cells from the indicated groups (n = 5). **(G)** Representative immunohistochemistry (IHC) images of tumor sections stained with Ki-67 or HK2 antibodies. Scale bar: 100 μm. One-way ANOVA, Dunnett's multiple comparisons test (A); Student's t-test (B-D). ****P < 0.001. Data are presented as mean ± SD.

The above findings suggested that ANXA2P1 was upregulated in AGS cells upon glucose deprivation (Figure 1A1). To verify this, we treated GC cells with 1.0 mmol L^-1^ glucose for 0, 6, 12, and 24 h. The results showed that glucose deprivation could induce ANXA2P1 overexpression time-dependently in MKN-74 and AGS cells (Figure 2A and S4A). Moreover, we showed that overexpression of ANXA2P1 significantly upregulated the glucose uptake and lactate generation in MKN-74 and HGC-27 cells (Figure 2B1, 2C1, S4B, and S4C). Conversely, the glucose consumption and lactate generation were reduced following ANXA2P1 knockdown in AGS and MKN-45 cells (Figure 2B2, 2C2, S4B, and S4C).

Furthermore, we employed a fluorescent d-glucose analog 2-[N-(7-nitrobenz-2-oxa-1,3-diazol-4-yl) amino]-2-deoxy-D-glucose (2-NBDG) to quantify glucose incorporation in living cells via flow cytometry 19. The outcomes displayed that overexpression of ANXA2P1 significantly increased 2-NBDG uptake, while its knockdown reduced uptake in GC cells (Figure 2D1-2). To further evaluate the role of ANXA2P1 in glycolysis, we measured glycolytic flux by assessing the extracellular acidification rate (ECAR) using the Seahorse Analyzer. The data illustrated that forced expression of ANXA2P1 markedly increased glycolytic flux, including glycolysis, glycolytic capacity, and glycolytic reserve, compared to the control (Figure 2E1 and S4D1). Conversely, knockdown of ANXA2P1 inhibited glycolytic flux of GC cells (Figure 2E2 and S4D2). These findings demonstrated a positive role of ANXA2P1 in the aerobic glycolysis of GC cells.

To substantiate this conclusion *in vivo*, MKN-74 cells with stable overexpression of ANXA2P1 (Lv-ANXA2P1) or control (Lv-control) were implanted into nude mice, followed by treatment with or without the glycolytic inhibitor 2-deoxy-D-glucose (2-DG). The tumor sizes in the ANXA2P1 group were considerably larger, while those in the ANXA2P1 + 2-DG-treated group were noticeably smaller than the control group (Figure 2F and S4E-F). Next, Hematoxylin and eosin (H&E) staining was conducted for the visualization of tumor in mouse tissues (Figure S4G). Immunohistochemical (IHC) staining indicated that the ANXA2P1 group exhibited significantly increased expression of proliferation marker Ki-67 and glycolysis marker HK2 compared with the control group, while the ANXA2P1 plus 2-DG group revealed reduced expression relative to the ANXA2P1 group (Figure 2G and S4H-I). Collectively, these outcomes suggest that ANXA2P1 elevates aerobic glycolysis in GC *in vitro* and *in vivo*.

### ANXA2P1 interacts with hnRNP F in GC cells

**Figure 3:**
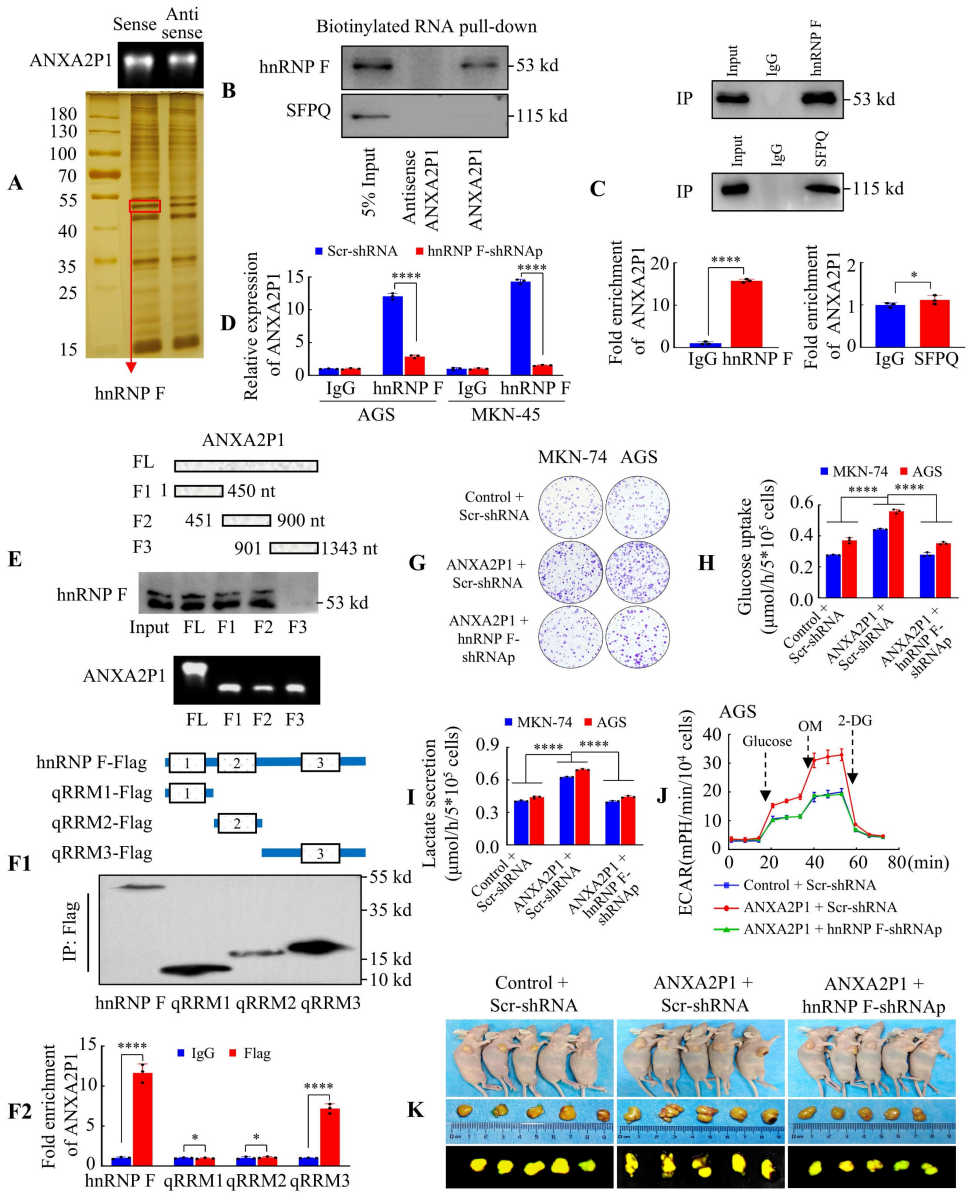
** ANXA2P1 specifically interacts with hnRNP F to facilitate growth and glycolytic metabolism in GC cells**. **(A)** Electrophoretic analysis of biotinylated ANXA2P1 probes and silver staining of proteins bound to ANXA2P1. **(B)** Western blotting of ANXA2P1-interacting proteins from RNA pull-down assays.** (C)** RNA immunoprecipitation (RIP) demonstrates a specific interaction between ANXA2P1 and hnRNP F in AGS cells, while no such interaction was observed with SFPQ.** (D)** RIP assays were performed in AGS and MKN-45 cells after hnRNP F knockdown to validate hnRNP F binding to ANXA2P1. **(E)** Western blotting of hnRNP F in AGS cells pulled down using *in vitro* transcribed, biotinylated RNAs corresponding to different ANXA2P1 constructs. Top: Schematic representation of the ANXA2P1 truncations used. Bottom: Verification of RNA probes by nucleic acid electrophoresis. **(F1/2)** Characterization of hnRNP F fragments interacting with ANXA2P1 by RIP assays. (F1) Top: Schematic representation of the hnRNP F fragments. Bottom: Verification of full-length or truncated hnRNP F by Western blotting using a flag antibody. (F2) RT-qPCR analysis of ANXA2P1 enrichment after RIP assay with anti-flag antibody. **(G)** Colony formation assays were performed in the indicated GC cells. **(H-I)** Glucose consumption (H) or lactate level (I) was measured in the supernatants of AGS and MKN-74 cells. **(J)** ECAR was measured in the indicated AGS cells.** (K)** Representative images of subcutaneous tumors of mice injected with the indicated AGS cells (n = 5). Student's t-test (C-F); one-way ANOVA, Dunnett's multiple comparisons test (H-I). *P > 0.05 and ****P < 0.001. Data are presented as mean ± SD.

Pseudogenes may exert regulatory functions through various mechanisms, including modulating their parental genes or interacting with proteins 7, 8, 20. A previous study showed that ANXA2P1 regulates parent gene ANXA2 expression in HCC cells 9. Thus, we identified proteins related to ANXA2P1 via RNA pull-down assays in GC cells. First, SDS-PAGE electrophoresis followed by silver staining was utilized to separate and visualize the ANXA2P1-binding proteins in AGS cells, and mass spectrometry (MS) was employed to analyze them (Figure 3A). The results indicated that 128 proteins interacted with ANXA2P1 (Table S3A). Subsequently, these 128 proteins were subjected to Gene Ontology (GO) enrichment analyses via the STRING database (https://cn.string-db.org/), and 18 significantly enriched molecular functions were identified. The top-ranked category was RNA binding, which included 50 proteins such as RBM22, SFPQ, hnRNP F and RBM8A, among others. Given that ANXA2P1 was identified as a glycolysis-associated pseudogene, we focused on RNA-binding proteins (RBPs) that have been previously reported to participate in glycolytic regulation. Among the proteins within the RNA-binding category, only two proteins, SFPQ and hnRNP F, have been experimentally reported to participate in glycolysis (Table S3B) 21, 22. These two proteins were therefore selected as candidate RBPs for further analysis. In parallel, RNA-protein interaction prediction using the RPISeq database suggested high interaction probabilities for both ANXA2P1-SFPQ and ANXA2P1-hnRNP F, as assessed by Random Forest and Support Vector Machine algorithms (Figure S5A1-2).

We then performed experimental validation of these two candidate proteins. RNA pull-down followed by Western blot illustrated that hnRNP F, but not SFPQ, bound with ANXA2P1 (Figure 3B). Furthermore, the RNA-immunoprecipitation (RIP) assay in AGS cells revealed an endogenous interaction between ANXA2P1 and hnRNP F, but not SFPQ (Figure 3C). To verify again, we discovered that hnRNP F suppression in AGS and MKN-45 cells significantly lessened ANXA2P1 enrichment by hnRNP F antibody in the RIP assay compared with scr-shRNA group (Figure 3D). This outcome illustrated that hnRNP F protein was physically interacted with ANXA2P1 *in vitro*.

Previous studies showed that hnRNP F regulates glycolysis in breast cancer cell lines 22. To further determine whether hnRNP F influences glycolysis in GC, we initially examined the effectiveness of hnRNP F upregulation and downregulation (Figure S5B1-2). Then, we evaluated the functional influence of hnRNP F on glycolysis in GC cells. Figure S5C-D illustrate that hnRNP F knockdown significantly suppressed glucose uptake and lactate generation.

To detect the hnRNP F-binding region on ANXA2P1, we constructed four ANXA2P1 fragments (F), as illustrated in the schematic diagram. Subsequently, RNA pull-down assays and Western blot revealed that the ANXA2P1-specific binding sequence was located between 1 and 900 nucleotides (F1 and F2) (Figure 3E). The hnRNP F protein comprised three key domains: quasi-RNA recognition motif (qRRM) 1 to qRRM3 (qRRM1, 13 ~ 85 aa; qRRM2, 111 ~ 188 aa; qRRM3, 289 ~ 366 aa) (Figure 3F1). To better determine which domain is responsible for ANXA2P1 binding, we performed RIP assays using FLAG-tagged full-length or truncated forms of hnRNP F and demonstrated that the hnRNP F qRRM3 domain (289 ~ 366 aa) was essential for ANXA2P1 binding (Figure 3F2). Collectively, our data indicate that ANXA2P1 physically interacts with hnRNP F protein in GC cells.

### ANXA2P1 cooperates with hnRNP F to facilitate growth and glycolytic metabolism in GC cells

Considering the critical function of ANXA2P1 in GC cell growth and aerobic glycolysis, we investigated the potential regulatory relationship between ANXA2P1 and hnRNP F in this process. To explore this possibility, we initially investigated whether ANXA2P1 influences hnRNP F expression. Results showed that upregulation or suppression of ANXA2P1 affected both protein and mRNA levels of hnRNP F (Figure S6A and B). Second, CCK-8 and CFA assays illustrated that ANXA2P1 overexpression enhanced the growth and colony formation capability of HGC-27 and MKN-74 cells, while hnRNP F knockdown partially reversed these effects (Figure 3G and S6C-D). Furthermore, glycolysis metabolism assays revealed that hnRNP F downregulation dramatically blocked ANXA2P1-induced glucose uptake and lactate production (Figure 3H-I). Notably, knockdown of hnRNP F abolished the increase in ECAR after overexpression of ANXA2P1 (Figure 3J and S6E).

To further confirm these results *in vivo*, subcutaneous xenograft tumors were generated in BALB/c nude mice using AGS cells stably expressing Lv-Control + Scr-shRNA, Lv-ANXA2P1 + Scr-shRNA, or Lv-ANXA2P1 + hnRNP F-shRNAp (shRNApool), respectively. Results showed that mice injected with ANXA2P1-overexpressing cells developed significantly larger subcutaneous tumors than those injected with control cells, whereas hnRNP F knockdown effectively abrogated the ANXA2P1-induced tumor growth (Figure 3K and S6F-G). Next, histological analysis was conducted to validate the existence of cancerous tissue. Subsequently, we employed IHC for Ki-67 and HK2 to assess cell proliferation and glycolysis markers. Both Ki-67 and HK2 levels in the ANXA2P1 group were found to be increased compared to the control, while hnRNP F-shRNAp attenuated these ANXA2P1-induced elevations (Figure S6H-J).

The results indicated that ANXA2P1 combined with hnRNP F promotes proliferation and glycolytic metabolism in GC cells.

### ANXA2P1 and hnRNP F interact with HK2 3′UTR to modulate HK2 expression at post-transcriptional level

**Figure 4:**
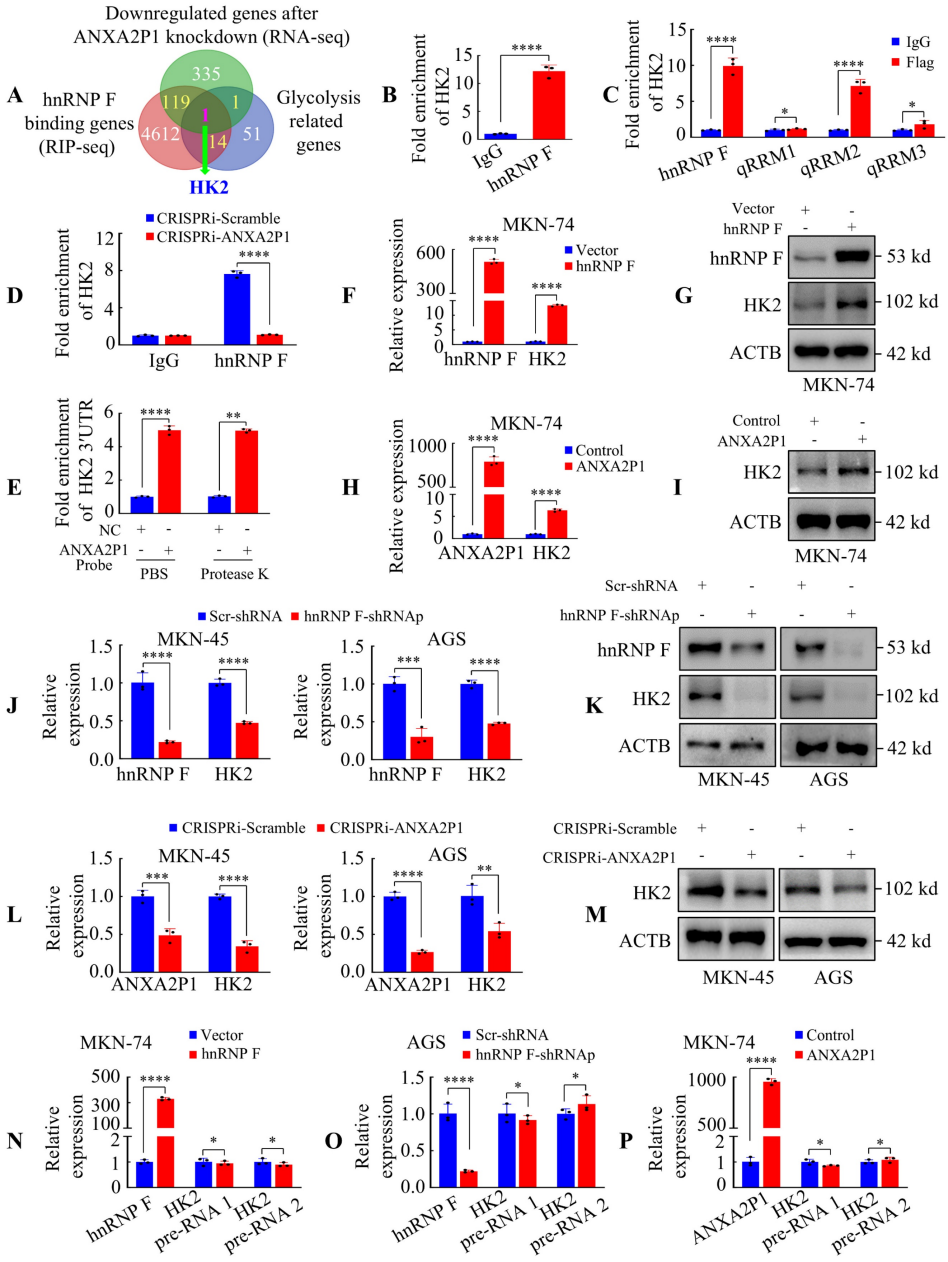
** ANXA2P1 and hnRNP F interact with HK2 3′UTR to modulate HK2 expression at post-transcriptional level. (A)** A Venn diagram displays the overlap among genes downregulated by ANXA2P1 knockdown (RNA-seq), hnRNP F-binding targets (RIP-seq), and glycolysis-related genes from the Kyoto encyclopedia of genes and genomes database (https://www.genome.jp/kegg/). **(B)** RIP assay in AGS cells indicated that hnRNP F bound directly to HK2 mRNA. **(C)** RIP assay was performed in AGS cells transfected with flag-tagged full-length or truncated hnRNP F constructs. RT-qPCR was used to measure the enrichment of HK2. **(D)** Assessment of RIP assays detecting HK2 mRNA retrieved by hnRNP F antibody or IgG in AGS cells transduced with CRISPRi-ANXA2P1 or CRISPRi-Scramble.** (E)** RAP analysis was performed to assess the relative enrichment of HK2 3′UTR by 5'biotin-labeled DNA probes specifically targeted ANXA2P1, with or without protease K treatment in substrate. NC negative control. **(F-I)** RT-qPCR and Western blotting analyses of the effects of hnRNP F (F-G) or ANXA2P1 (H-I) overexpression on HK2 mRNA and protein levels. **(J-M)** RT-qPCR and Western blotting analyses of the effects of hnRNP F (J-K) or ANXA2P1 (L-M) knockdown on HK2 mRNA and protein levels. **(N-P)** RT-qPCR analysis of HK2 pre-mRNA levels in GC cells after indicated treatment. Student's t-test. *P > 0.05, **P < 0.05, ***P < 0.01, and ****P < 0.001. Data are presented as mean ± SD.

Given that the RNA-binding protein hnRNP F is involved in both precursor mRNA (pre-mRNA) processing and mature mRNA stability through direct RNA interactions 23-25, we speculated that the interaction between ANXA2P1 and hnRNP F might affect the processing or stability of specific target mRNAs. To profile mRNA targets regulated by ANXA2P1, we performed RNA sequencing (RNA-seq) following ANXA2P1 knockdown in AGS cells (|FC| > 1.2). In parallel, RNA immunoprecipitation sequencing (RIP-seq) was conducted in AGS cells to identify hnRNP F-interacting genes. RNA-seq analysis revealed 456 downregulated genes, including hnRNP F and HK2, and 176 upregulated genes (Table S4), while RIP-seq identified 4746 potential hnRNP F-binding genes, including HK2 (Table S5). Overlapping the RNA-seq and RIP-seq datasets with glycolysis-related genes from the KEGG database (https://www.genome.jp/kegg/) (Table S6) identified HK2 as the only overlapping gene (Figure 4A).

The RIP RT-qPCR assay was conducted to assess the interaction between hnRNP F and HK2 mRNA (Figure 4B). We subsequently aimed to identify the domain of hnRNP F responsible for interaction with HK2 mRNA and demonstrated that the hnRNP F-qRRM2 domain (111 ~ 188 aa) was essential for this interaction (Figure 4C). Furthermore, we investigated whether ANXA2P1 was involved in the interaction between hnRNP F and HK2 mRNA. RIP assay results showed that HK2 mRNA precipitated by hnRNP F was markedly decreased in CRISPRi-ANXA2P1 cells compared with CRISPRi-Scramble (Figure 4D), indicating that ANXA2P1 is essential for the interaction between hnRNP F and HK2.

In addition**,** the IntaRNA algorithm predicted complementary base-pairing between ANXA2P1 and the HK2 3′UTR (Figure S7A). We applied RNA antisense purification (RAP) assay to demonstrate the interaction between ANXA2P1 and HK2 3′UTR. Results showed that protease K treatment to remove the proteins from the substrate did not affect the HK2 3′UTR level pulled down by ANXA2P1 probes (Figure 4E). Thus, we verified that the interaction between ANXA2P1 and HK2 3′UTR was independent of proteins.

We next assessed the expression levels of HK2 while varying hnRNP F or ANXA2P1 levels. Figure 4F-I illustrate that forced expression of hnRNP F or ANXA2P1 magnified HK2 mRNA and protein levels in MKN-74 cells, whereas knockdown of hnRNP F or ANXA2P1 decreased HK2 mRNA and protein expression in AGS and MKN-45 cells (Figure 4J-M). Nevertheless, hnRNP F or ANXA2P1 manipulation did not influence HK2 transcription, as demonstrated by unaffected HK2 pre-mRNA levels **(**Figure 4N-P and S7B-D). These outcomes illustrate that HK2 is regulated by hnRNP F or/and ANXA2P1 at the post-transcriptional level.

### ANXA2P1 and hnRNP F coregulate HK2 mRNA stability by promoting its 3′UTR shortening in GC cells

**Figure 5:**
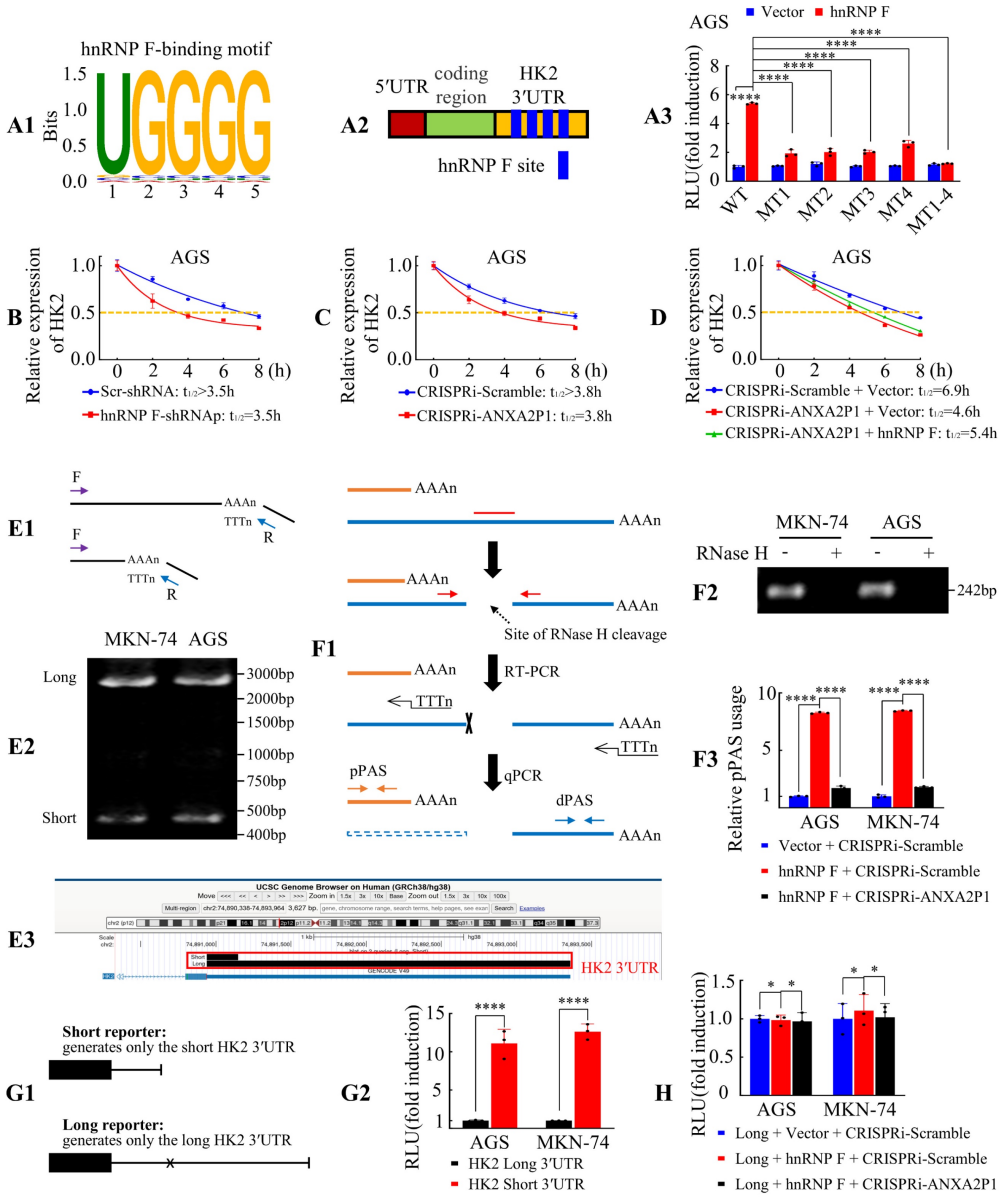
** ANXA2P1 and hnRNP F collaboratively regulate HK2 mRNA stability by promoting its 3′UTR shortening in GC cells. (A1-3)** The hnRNP F-binding motif was identified, as illustrated in (A1). Schematic representation of the HK2 3′UTR displaying putative hnRNP F-binding site (A2). Relative luciferase activities of HK2 3′UTR in AGS cells after indicated treatment (A3). WT, Wild type; MT, Mutation. **(B-C)** Assessment of HK2 mRNA half-life (T1/2) following knockdown of hnRNP F (B) or ANXA2P1 (C) in AGS cells. **(D)** Assessment of the cooperative effects of ANXA2P1 and hnRNP F on HK2 mRNA half-life. **(E1-3)** Identification of HK2 3′UTR isoforms by 3′RACE. Schematic of the 3′RACE strategy (E1) and agarose gel showing long and short HK2 3′UTR isoforms in MKN-74 and AGS cells (E2). Sequencing of 3′RACE products mapped to the HK2 3′UTR, visualized in the UCSC Genome Browser (E3). **(F1-3)** RHAPA analysis of HK2 3′UTR. Schematic of the RHAPA workflow (F1). Agarose gel confirming RNase H cleavage (F2) and quantification of proximal versus distal polyadenylation site usage by RHAPA following indicated treatment (F3). RHAPA, RNase H alternative polyadenylation assay; pPAS, proximal polyadenylation signal; dPAS, distal polyadenylation signal. **(G1-2)** Luciferase reporter assays. Schematic of short and long reporters (G1). Dual-luciferase results for indicated reporters (G2). **(H)** Dual-luciferase assays after indicated transfection. One-way ANOVA, Dunnett's multiple comparisons test (A, F, and H); Student's t-test (G). **P < 0.05, ***P < 0.01, and ****P < 0.001. Data are presented as mean ± SD.

Motif enrichment analysis based on the above RIP-seq data revealed a G-rich element containing the UGGGG sequence (Figure 5A1), consistent with previous reports 26 and RBP motif databases such as oRNAment (http://rnabiology.ircm.qc.ca/ oRNAment) and ATtRACT (https://dblp.org/rec/journals/biodb/ GiudiceSFP16.html). Among the enriched targets (Table S5), ANXA2P1 was identified as a prominent hnRNP F-bound transcript containing the UGGGG motif, in agreement with RNA pull-down results showing that the hnRNP F-binding region resides within the F1/2 fragments of ANXA2P1 (Figure 3E). Meanwhile, hnRNP F was enriched on HK2 mRNA, mainly at the 3′UTR (chr2: 74890819 to 74893421), which contains four hnRNP F-binding sites with the UGGGG motif (Figure 5A2) as revealed by RIP-seq analysis. ANXA2P1 is mainly localized in the nucleus (Figure 1D and E), and hnRNP F has been reported to regulate mRNA stability by facilitating 3′UTR processing and polyadenylation in the nucleus 23. Given these findings, we hypothesized that ANXA2P1 and hnRNP F collaboratively stabilize HK2 mRNA by interacting with HK2 3′UTR.

We then performed luciferase reporter assays to validate this interaction by cloning the full-length HK2-WT-3′UTR and four site-specific mutants into dual-luciferase reporter vectors. Observations illustrated that hnRNP F overexpression significantly elevated luciferase activity of the wild-type reporter, while all four mutants (MT1-4) showed markedly reduced activity driven by the HK2 3′UTR in AGS and MKN-45 cells (Figure 5A3 and S8A), suggesting that these sites are critical for hnRNP F binding and regulation. These outcomes illustrated that hnRNP F bound to the HK2 3′UTR and may enhance its mRNA stability by preventing degradation.

To explore whether ANXA2P1 was involved in hnRNP F-mediated HK2 mRNA metabolism, luciferase reporter assays were performed, and the results illustrated that ANXA2P1 knockdown attenuated the hnRNP F-induced increase in luciferase activity driven by the HK2-WT-3′UTR in GC cells (Figure S8B). These findings suggested that ANXA2P1 facilitated the interaction between HK2 3′UTR and hnRNP F.

Accumulating evidence indicates that hnRNPs participate in the regulation of RNA stability 27. To further validate that ANXA2P1 and hnRNP F collaboratively regulate HK2 mRNA stability, cells were subsequently exposed to Actinomycin D to inhibit novel RNA synthesis and HK2 mRNA levels were examined to assess its stability. Results showed that hnRNP F knockdown accelerated HK2 mRNA decay (Figure 5B and S8C), while hnRNP F overexpression slowed its degradation (Figure S8D) in GC cells, implying that hnRNP F stabilized HK2 mRNA. ANXA2P1 suppression also augmented HK2 mRNA degradation (Figure 5C and S8E), whereas upregulation of ANXA2P1 inhibited its decay (Figure S8F). Importantly, overexpression of hnRNP F partially reversed the destabilizing effect caused by ANXA2P1 suppression (Figure 5D and S8G), implying an indispensable role of ANXA2P1 in facilitating the interaction between hnRNP F and the HK2 3′UTR.

Alternative cleavage and polyadenylation (APA) involves the selection of alternative cleavage and polyadenylation sites to generate mRNA isoforms with different 3'UTR lengths. In tumor cell lines, APA often shifts toward proximal site selection, resulting in shortened 3′UTRs. By losing binding sites for repressive microRNAs, these shortened variants usually exhibit enhanced stability 28. Given that hnRNP F has been reported to facilitate 3′UTR cleavage and polyadenylation in the nucleus 23, we next investigated whether ANXA2P1 and hnRNP F coregulate HK2 mRNA stability through APA. To characterize the 3′-end architecture of HK2 transcripts, we performed 3′RACE followed by sequencing. Two distinct HK2 mRNA isoforms with different 3′UTR lengths were identified, demonstrating the presence of APA in HK2 3′UTR (Figure 5E1-3). These cleavage sites were consistent with PolyASite annotations (https://polyasite.unibas.ch). We designated the cleavage and polyadenylation site that generates the short 3′UTR isoform as the proximal site (chr2:74,891,162), and the downstream site that generates the long 3′UTR isoform as the distal site (chr2:74,893,307). As shown in Figure 5A3 and Figure S8A, the hnRNP F binding region is located downstream of the proximal cleavage and polyadenylation site within the HK2 3′UTR. This close spatial proximity supports that hnRNP F may enhance usage of the proximal cleavage and polyadenylation site, thereby promoting production of the short HK2 3′UTR isoform.

To functionally validate this hypothesis, we performed RHAPA, a RT-qPCR-based method specifically developed to quantify APA events 16, 17. Results showed that hnRNP F overexpression markedly increased the abundance of the short 3′UTR isoform, whereas ANXA2P1 knockdown significantly attenuated this effect (Figure 5F1-3). These findings indicate that ANXA2P1 cooperates with hnRNP F to promote usage of the proximal cleavage and polyadenylation site, thereby generating the short HK2 3′UTR isoform. To compare the stability of HK2 transcripts with different 3′UTR lengths, we constructed dual-luciferase reporters that exclusively generate either the short or the long HK2 3′UTR isoform, whereas the WT reporter described above produces both isoforms. In the long 3′UTR reporter, proximal polyadenylation signals annotated in the PolyASite database were mutated to prevent usage of the proximal site. Reporter assays showed that the short 3′UTR construct exhibited significantly higher luciferase activity than the long 3′UTR construct, indicating that the short 3′UTR enhanced transcript stability (Figure 5G1-2). Notably, alteration of hnRNP F or ANXA2P1 expression levels did not affect luciferase activity in cells transfected with the long 3′UTR reporter (Figure 5H), demonstrating that the ability of ANXA2P1 and hnRNP F to regulate HK2 mRNA stability depends on intact proximal polyadenylation signals.

Collectively, these data demonstrate that ANXA2P1 cooperates with hnRNP F to promote usage of the proximal cleavage and polyadenylation site, leading to the generation of a shorter 3′UTR isoform and thereby enhancing HK2 mRNA stability in GC cells.

### Co-regulation of ANXA2P1 and hnRNP F modulates HK2 expression to promote GC cell proliferation and glycolysis

To investigate the contribution of HK2 to ANXA2P1- and hnRNP F-induced GC growth and glycolysis, we established seven experimental groups: group A, CRISPRi-Scramble + Scr-shRNA + Vector; group B, CRISPRi-Scramble + Scr-shRNA + HK2; group C, CRISPRi-ANXA2P1 + Scr-shRNA + Vector; group D, CRISPRi-ANXA2P1 + Scr-shRNA + HK2; group E, CRISPRi-Scramble + hnRNP F-shRNAp + Vector; group F, CRISPRi-Scramble + hnRNP F-shRNAp + HK2; and group G, CRISPRi-ANXA2P1 + hnRNP F-shRNAp + HK2. First, we estimated the transfection efficacy of HK2 by RT-qPCR and Western blot (Figure S9A-B). Results illustrated that compared with the control cells (group A), knockdown of ANXA2P1 (group C) or hnRNP F (group E) significantly suppressed cell proliferation, glucose uptake, lactate production, and ECAR. However, the suppressive effect was dramatically reversed when we introduced HK2 into CRISPRi-ANXA2P1 (group D) or hnRNP F-shRNAp (group F) expressing cells. Moreover, in group G, we transfected HK2 into GC cells with simultaneous ANXA2P1 knockdown and hnRNP F knockdown. Results showed that HK2 overexpression significantly diminished the inhibitory effects on cell growth and glycolytic phenotypes caused by ANXA2P1-silencing and hnRNP F-silencing (Figure S9C-I).

Taken together, these results illustrate that co-modulation of ANXA2P1 and hnRNP F regulates HK2 expression to facilitate growth and aerobic glycolysis in GC cells.

### ANXA2P1 is transcriptionally driven by the c-Myc/HK2 complex in GC cells

**Figure 6:**
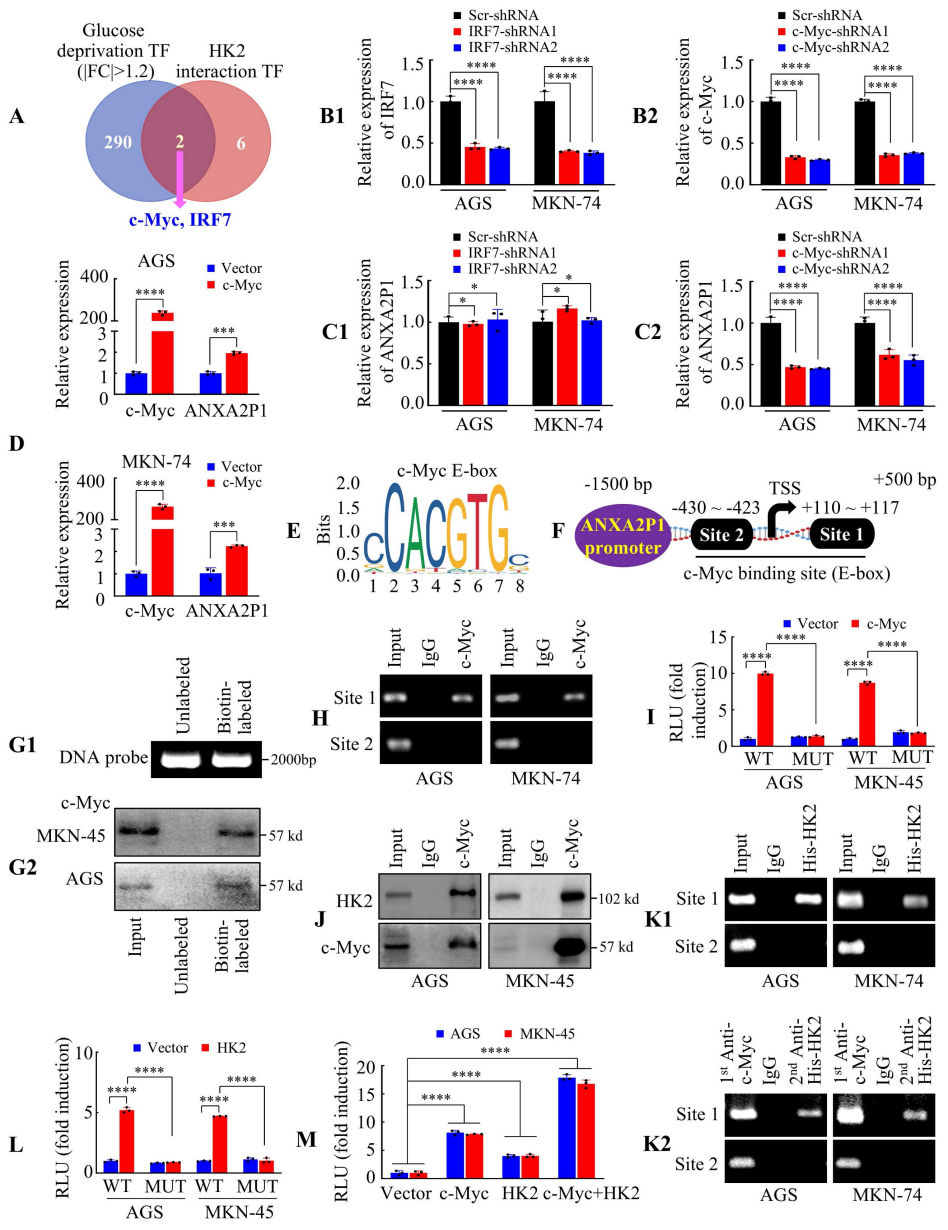
** ANXA2P1 is transcriptionally regulated by the c-Myc/HK2 complex in GC cells. (A)** A Venn diagram illustrates the overlap of transcription factors (TF, annotated using JASPAR database) between HK2-interacting proteins from BioGRID and differentially expressed genes under glucose deprivation. **(B1/2-C1/2)** RT-qPCR analysis of IRF7 (B1 and C1) or c-Myc (B2 and C2) knockdown efficiency and their effects on ANXA2P1 expression in GC cells.** (D)** RT-qPCR analysis of c-Myc overexpression efficiency and its effect on ANXA2P1 expression in GC cells.** (E)** JASPAR predicts the c-Myc binding motif.** (F)** Schematic diagram of the ANXA2P1 promoter indicating two potential c-Myc-binding sites. **(G1/2)** Electrophoretic analysis of unlabeled and biotin-labeled DNA probes (G1). Western blotting indicated c-Myc was pulled down by the ANXA2P1 promoter probe in AGS and MKN-45 cells (G2). **(H)** ChIP assays demonstrated the binding of c-Myc to ANXA2P1 promoter sites in GC cells. **(I)** Relative luciferase activity of wildtype (WT) or site 1 mutant (MUT) ANXA2P1 promoter luciferase reporter plasmids in cells transfected with c-Myc or vector. **(J)** Coimmunoprecipitation detecting the endogenous interaction between HK2 and c-Myc in AGS and MKN-45 cells. Immunoprecipitates obtained with anti-c-Myc antibody or control IgG were confirmed by Western blotting. **(K1/2)** Enrichment of the ANXA2P1 promoter region containing c-Myc binding sites 1 and 2 in GC cells was determined by the ChIP-PCR (K1) and Re-ChIP-PCR (K2) assays using the indicated antibodies. **(L)** Relative luciferase activity of WT or site 1 MUT ANXA2P1 promoter luciferase reporter plasmids in cells transfected with HK2 or vector. **(M)** Luciferase assays were performed to assess ANXA2P1 promoter activity in AGS and MKN-45 cells with the indicated transfections. One-way ANOVA, Dunnett's multiple comparisons test (B, C, and I-M); Student's t-test (D). *P > 0.05, ***P < 0.01, and ****P < 0.001. Data are presented as mean ± SD.

Previous investigations illustrated that nuclear HK2 interacts with transcription factor proteins and functions as a transcriptional co-regulator 29, 30. To further explore transcription factors potentially regulating ANXA2P1, we integrated protein interaction data from BioGRID with gene expression data from microarray analysis. According to BioGRID (https://thebiogrid.org/), HK2 interacts with 120 proteins, including eight transcription factors based on the JASPAR database (https://jaspar.elixir.no/). Microarray analysis of protein-coding genes in AGS cells under glucose deprivation revealed 9493 differentially expressed genes (|FC| > 1.2), comprising 4412 upregulated genes (including c-Myc) and 5081 downregulated genes (including IRF7), among which 292 were transcription factors (Table S7). Venn diagram analysis revealed two overlapping candidates: IRF7 and c-Myc (Figure 6A).

We subsequently examined whether IRF7 or c-Myc modulates ANXA2P1 expression. The knockdown efficiency was confirmed in GC cells (Figure 6B1-2). Results illustrated that IRF7 suppression had no influence on ANXA2P1 expression (Figure 6C1), whereas silencing c-Myc significantly reduced its expression (Figure 6C2). Conversely, c-Myc overexpression upregulated ANXA2P1 expression in both AGS and MKN-74 cells (Figure 6D). Hence, our data illustrated that c-Myc expression is positively linked to ANXA2P1 expression levels, suggesting c-Myc may transcriptionally regulate ANXA2P1.

Considering c-Myc identifies E-box elements (Figure 6E) (E-boxes: 5′-CANNTG-3′) 31, 32, we scanned the promoter of ANXA2P1 using JASPAR and identified two potential c-Myc binding sites: +110 ~ +117 bp and -430 ~ -423 bp (Figure 6F). We first designed a biotinylated DNA probe encompassing the binding sites within the ANXA2P1 promoter region (-1500 ~ +500 bp) for DNA pull-down experiments (Figure 6G1). Results demonstrated that c-Myc could be pulled down by the ANXA2P1 promoter probe (Figure 6G2). Subsequently, chromatin immunoprecipitation (ChIP) assay was performed to investigate whether c-Myc binds directly to the ANXA2P1 promoter. Results showed that site 1 of the ANXA2P1 promoter, but not site 2, was significantly enriched in samples immunoprecipitated with an anti-c-Myc antibody, whereas no enrichment was observed with the IgG control (Figure 6H). In addition, we cloned the full-length ANXA2P1 promoter and a site1 mutant promoter into luciferase reporter plasmids. Results showed that c-Myc overexpression significantly elevated the activity of the wild-type ANXA2P1 promoter, but not the mutant promoter (Figure 6I), illustrating that the first binding site was critical for c-Myc-induced ANXA2P1 transactivation.

To further investigate whether the c-Myc-HK2 complex is tethered to the ANXA2P1 promoter, we performed coimmunoprecipitation (Co-IP) of endogenous proteins, which confirmed an interaction between c-Myc-HK2 proteins in GC cells (Figure 6J). Meanwhile, ChIP and Re-ChIP assays showed that both c-Myc and HK2 bind to the ANXA2P1 promoter at site 1 (Figure 6K1-2). Moreover, HK2 overexpression also elevated the promoter activity of ANXA2P1 in GC cells (Figure 6L). To further validate the cooperative effect, GC cells were co-transfected with c-Myc and HK2, either individually or in combination, along with the ANXA2P1 promoter luciferase reporter. Luciferase assays demonstrated that overexpression of either c-Myc or HK2 alone significantly increased ANXA2P1 promoter activity, while their combined overexpression exerted an even greater activation (Figure 6M). These studies indicated that HK2 might interact with c-Myc to modulate the transcription of ANXA2P1.

Taken together, these data illustrate that ANXA2P1 is transcriptionally promoted by the c-Myc/HK2 complex in GC cells.

### ANXA2P1 is modulated by c-Myc/HK2 complex to promote GC proliferation and aerobic glycolysis

To explore the potential function of ANXA2P1 modulated by c-Myc/HK2 complex, we first established GC cell lines with stable c-Myc overexpression and knockdown, which were verified by Western blot analysis (Figure S10A1-2). Next, the experiments were assigned to seven groups: group A, Scr-shRNA + control; group B, c-Myc-shRNAp + control; group C, Scr-shRNA + ANXA2P1; group D, c-Myc-shRNAp + ANXA2P1; group E, c-Myc-shRNAp + Scr-shRNA + control; group F, c-Myc-shRNAp + HK2-shRNAp + control; and group G, c-Myc-shRNAp + HK2-shRNAp + ANXA2P1. Results indicated that group B (c-Myc knockdown) suppressed proliferation and glycolysis compared to group A (control), while group D (c-Myc knockdown + ANXA2P1 overexpression) rescued these effects. Furthermore, group F (c-Myc-shRNAp + HK2-shRNAp + control) simultaneously inhibited GC growth and glycolysis more profoundly than group E (c-Myc knockdown) alone, and this inhibition was partially reversed by group G (c-Myc-shRNAp + HK2-shRNAp + ANXA2P1), as shown by CCK-8, CFA, ECAR, glucose uptake, and lactate production assays (Figure S10B-F).

Furthermore, AGS cells stably expressing the constructs of the seven experimental groups described above were subcutaneously implanted into nude mice. We found that the tumor weight and volume trends were consistent with the *in vitro* findings (Figure S11A-C). Histological staining, including H&E staining and IHC assay for Ki-67 and HK2, further validated tumor formation and proliferative activity in xenograft tissues (Figure S11D-H).

Taken together, these data show that ANXA2P1 is modulated by the c-Myc/HK2 complex to promote GC growth and glycolysis.

### Validation of ANXA2P1, hnRNP F, HK2 and c-Myc expression in GC specimens

**Figure 7:**
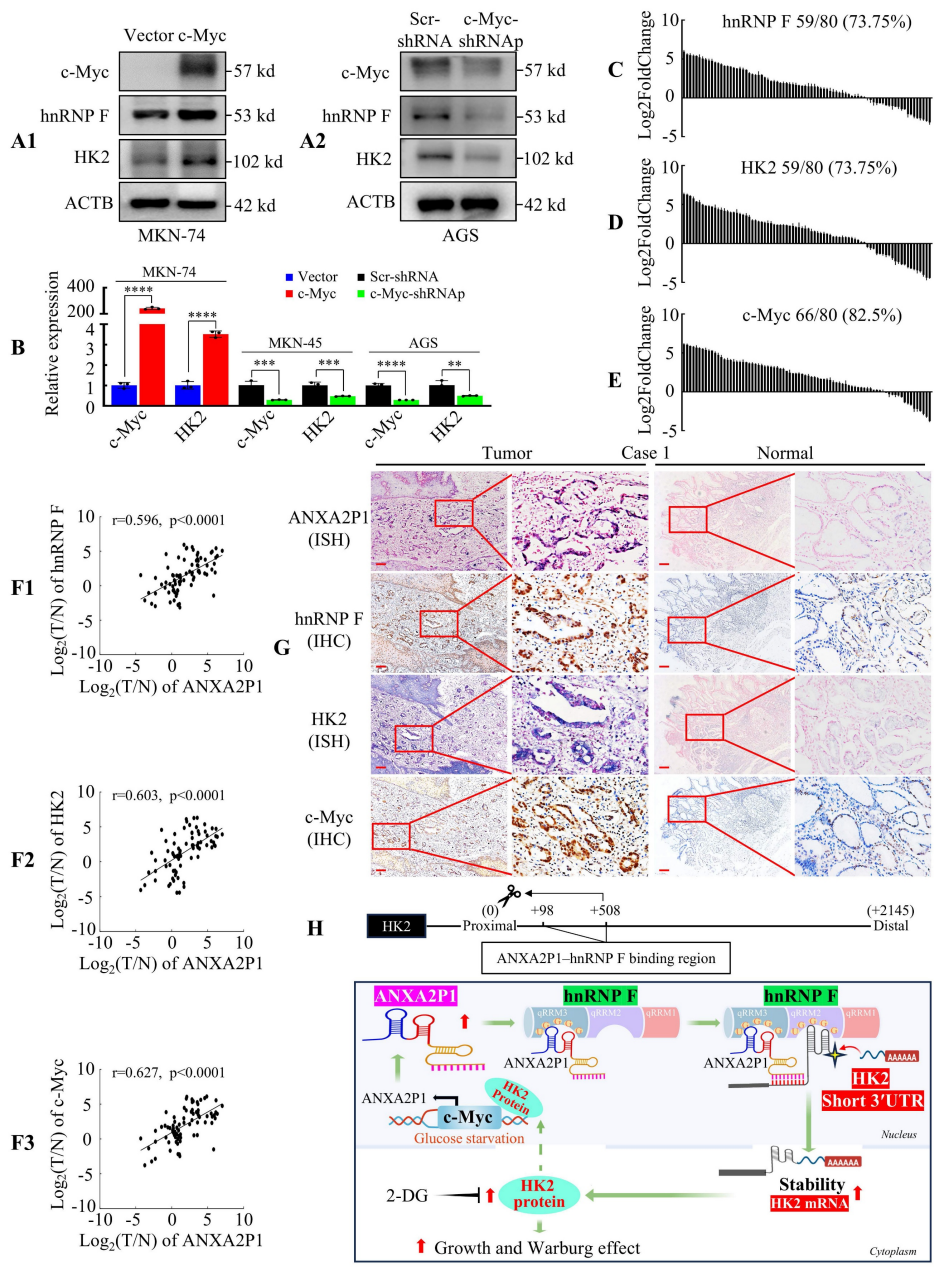
** ANXA2P1 expression levels are positively correlated with c-Myc, hnRNP F, and HK2 in GC. (A1/2)** Western blotting was performed to assess the effects of c-Myc overexpression (A1) or knockdown (A2) on the expression of hnRNP F and HK2 in GC cells. **(B)** RT-qPCR analysis of HK2 mRNA levels following c-Myc overexpression or knockdown.** (C-E)** Waterfall plots display the relative expression levels of hnRNP F (C), HK2 (D), and c-Myc (E) in 80 paired GC and adjacent normal tissues, as measured by RT-qPCR analysis**. (F1-3)** Correlation analyses between ANXA2P1 and hnRNP F (F1), or HK2 (F2), or c-Myc (F3) expression in 80 paired GC tissues.** (G)** Representative IHC and ISH images illustrate expression of the indicated genes in GC and adjacent normal tissues. Scale bar: 100 µm. Student's t-test (B); Pearson's correlation analysis (F). Data are presented as mean ± SD. **P < 0.05, ***P < 0.01, and ****P < 0.001.** (H)** Schematic representation of the function and potential mechanism of ANXA2P1 in GC.

Next, we investigated the relationship among c-Myc, hnRNP F, and HK2 expression levels. Western blot and RT-qPCR analysis in GC cells revealed that c-Myc overexpression significantly increased the expression of hnRNP F and HK2, while c-Myc knockdown markedly reduced their expression (Figure 7A1-2 and B). In addition, RT-qPCR analysis of 80 tumor samples revealed that hnRNP F, HK2, and c-Myc were frequently upregulated (Figure 7C-E and S12A-C). Furthermore, statistical analysis illustrated that ANXA2P1 expression showed a positive correlation with hnRNP F (Figure 7F1), HK2 (Figure 7F2), and c-Myc (Figure 7F3) expression in GC tissues.

We assessed the correlation between ANXA2P1 expression and clinicopathological parameters of GC individuals (Table S2A). To further assess the involvement of the ANXA2P1-hnRNP F-HK2/c-Myc axis, we explored the correlations between hnRNP F, HK2, c-Myc and diverse clinicopathological parameters. These outcomes revealed that overexpression of hnRNP F, HK2, or c-Myc was significantly positively correlated with lymph node metastasis (N-absent vs. N-present), distant metastasis (M-absent vs. M-present), and AJCC stage (I/II vs. III/IV) in GC patients. Moreover, HK2 and c-Myc, but not hnRNP F, exhibited a significant correlation with local tumor invasion (T1/2 vs. T3/4) (Table S2B-D).

Finally, ISH and IHC assays performed on serial sections confirmed that ANXA2P1, hnRNP F, HK2, and c-Myc showed significantly higher intensity in GC tissues than in the matched normal gastric mucosa from 30 patient samples, as exemplified in Figure 7G and S12D.

These results indicate that the ANXA2P1 level is positively related to hnRNP F, HK2, and c-Myc levels in GC patients.

## Discussion

Pseudogene-derived lncRNAs have been implicated in various pathological processes in tumor occurrence and development. Nevertheless, the biological functions and underlying mechanisms of ANXA2P1 in GC are still unknown. Here, we found that ANXA2P1 is considered an oncogenic pseudogene in GC and markedly facilitates GC cell growth and metastasis by mediating glycolysis. Mechanistically, ANXA2P1 specifically binds to RNA-binding protein hnRNP F, cooperatively enhancing the HK2 mRNA stability. In addition, ANXA2P1 expression levels are regulated by the c-Myc/HK2 complex. Collectively, the ANXA2P1-hnRNP F-HK2/c-Myc positive feedback loop promotes GC cell growth and glycolytic metabolism.

ANXA2P1 (annexin A2 pseudogene 1) is a processed pseudogene that is highly homologous to ANXA2, which encodes a calcium-dependent phospholipid-binding protein involved in the pathological processes of many neoplasms 33. Aberrant expression of ANXA2 has been documented in gastric adenocarcinoma 34, prostate carcinoma, pancreatic cancer, and breast cancer 35, suggesting its oncogenic potential. Recent studies have also shown that ANXA2P1 is transcribed in HCC cells 9. Nevertheless, the precise molecular function of ANXA2P1 in GC is undetermined. Herein, we discovered that ANXA2P1 was upregulated in GC tissues, and functional experiments showed that ectopic expression of ANXA2P1 modulated GC cell aggressiveness. Meanwhile, silencing ANXA2P1 significantly suppressed GC metastasis and invasion, indicating that ANXA2P1 may be an innovative therapeutic target for GC. In addition, a recent investigation reported that pseudogene-derived lncRNA WFDC21P could regulate glycolysis in hepatocarcinogenesis 14. Consistently, we demonstrated that ANXA2P1 overexpression facilitated aerobic glycolysis in GC cells by elevating glucose uptake, lactate generation, and ECAR. Conversely, the treatment of 2-DG exhibited a reverse effect in GC cells*.* Taken together, these outcomes suggest that ANXA2P1 is considered an oncogene in GC cells by facilitating aerobic glycolysis to promote proliferation.

HnRNP F is a 415-amino-acid RNA-binding protein, a member of the heterogeneous nuclear ribonucleoprotein (hnRNP) family. It plays an important role in regulating mRNA stability, including facilitating pre-mRNA 3′-end processing and polyadenylation in the nucleus, and modulating ARE-mediated mRNA decay in the cytoplasm 23, 24. In addition, hnRNP F has been implicated in alternative splicing 25. Collectively, hnRNP F functions in multiple post-transcriptional processes.

HnRNP F has oncogenic functions in various malignancies, with elevated expression observed in GC and documented roles in facilitating the growth and metastasis of malignant bladder cells 24, 36, 37. Besides, MS analysis revealed that hnRNP F physically binds to TPX2 in BC cells 37, and it has been reported to associate with lncRNA Mir100hg, facilitating its trafficking 38. Consistent with the above reports, we discovered that ANXA2P1 interacts with hnRNP F as demonstrated by MS, RNA pull-down, and RIP assays. Moreover, the oncogenic effects of ANXA2P1 overexpression were reversed by hnRNP F knockdown, suggesting that the function of ANXA2P1 in malignant biological processes is dependent on hnRNP F, which was consistent with our previous research that LINC01189 was associated with hnRNP F protein 36. Therefore, these findings suggest that ANXA2P1 cooperates with hnRNP F to promote proliferation and glycolytic metabolism in GC cells.

HK2, a vital metabolic enzyme of glycolysis, promotes glucose uptake in cells and facilitates the Warburg effect 39, 40. Aberrant upregulation of HK2 has been detected in various cancers and is linked to tumor progression. For instance, HK2 expression is significantly elevated and positively correlated with the Warburg effect and proliferation in GC 39. While HK2 functions primarily as a glycolytic enzyme contributing to cancer metabolism, emerging evidence suggests that its expression is under tight post-transcriptional control. For example, Jiang S *et al.* showed that HK2 mRNA expression is regulated by miR-143 at the post-transcriptional level 40. Certain cis-acting sequences and trans-acting factors contribute to the control of mRNA stability 41. hnRNP F contains three quasi-RRMs that preferentially bind to poly(G) sequences, particularly GGGA or DGGGD (where D denotes a U, G, or A nucleotide) motifs, and functions in modulating mRNA half-life 26, 42. However, the mechanism behind the HK2 mRNA stability affected by hnRNP F is still elusive. Here, we exhibited that hnRNP F was predominantly enriched at the HK2 3′UTR using RIP-seq analysis. This binding was validated by RIP, RT-qPCR, and luciferase reporter assays. Intriguingly, ANXA2P1 acts as a molecular link between hnRNP F and the HK2 3′UTR, thereby promoting hnRNP F-mediated proximal polyadenylation site usage of HK2, leading to the generation of a short 3′UTR isoform with enhanced stability. Functional rescue experiments confirmed that ANXA2P1 and hnRNP F cooperatively promote HK2 expression, leading to increased GC cell proliferation and aerobic glycolysis. Our findings align with the recent studies supporting that MIR100HG maintains the TCF7L2 mRNA stability by interacting with hnRNPA2B1 in colorectal cancer cells 43, highlighting a common mechanism whereby lncRNAs cooperate with RNA-binding proteins to affect mRNA stability in cancer progression. Thus, we identified ANXA2P1 and hnRNP F as positive regulators of HK2 mRNA to modulate biological function in GC cells.

C-Myc, a key oncoprotein, is well known for its critical roles in various biological processes, including energy metabolism 44, 45. It binds to canonical CACGTG or CATGTG motifs, thereby modulating the expression of numerous target genes and regulating multiple cellular functions 46, 47. For example, c-Myc binds to the PKM promoter to modulate aerobic glycolysis 47. Consistently, our bioinformatics analysis revealed that c-Myc is recruited to and binds to the promoter region of ANXA2P1 (-1500 ~ +500 bp). This finding was further validated by DNA pull-down, ChIP, and luciferase reporter assays, which demonstrates that c-Myc serves as a transcriptional activator of ANXA2P1 expression.

Some reports show that HK2 interacts with transcription factors to regulate biological processes 29, 30. For example, HK2 acts as a transcriptional regulator in the nucleus by interacting with transcription factor Nrf2 to control the promoter activity of XOR in glioma cells 29. Similarly, nuclear-localized HK2 interacts with the transcription factor MAX to modulate chromatin accessibility and maintain stemness in AML and normal hematopoietic stem/progenitor cells 30. Herein, we illustrated that HK2 and c-Myc formed a c-Myc/HK2 protein complex. Overexpression of either c-Myc or HK2, alone or in combination, increased ANXA2P1 expression and enhanced luciferase activity of the ANXA2P1 promoter in GC cells, indicating that the c-Myc/HK2 protein complex has a vital function in the transcriptional modulation of ANXA2P1. Similarly, Zeng J *et al.* reported that PCAF, CtBP1/2, and c-Myc form a complex that binds to the lncRNA CASC2 promoter 48, supporting the role of c-Myc-containing complexes as transcriptional regulators of noncoding genes. Furthermore, functional assays, including CCK-8, CFA, glucose consumption, lactate production, and ECAR, demonstrated that ANXA2P1 is modulated by the c-Myc/HK2 complex to promote GC cell growth and glycolysis. In line with these findings, the levels of c-Myc, HK2, and ANXA2P1 were significantly elevated in GC tissues compared to corresponding normal tissues, suggesting the potential oncogenic function of this axis in GC.

Our findings suggest important therapeutic implications for targeting the pseudogene ANXA2P1 in GC patients. Recent studies support the therapeutic potential of targeting specific oncogenic pseudogenes. For example, Ramesh-Kumar *et al.* reported that antisense LNA GapmeRs directed against the pseudogene RPSAP52 significantly reduced ovarian cancer tumor growth 49. In GC, glycolysis-targeting strategies have been actively explored and are considered a promising avenue. For instance, the glucose analogue 2-DG has been tested in GC models. However, the overall efficacy of glycolysis inhibition as a monotherapy remains suboptimal 50. Therefore, therapeutic strategies combining ANXA2P1-targeting approaches with glucose metabolism-targeted interventions may represent a promising direction to improve therapeutic efficacy in gastric cancer.

In summary, as depicted in our working model (Figure 7H), ANXA2P1 functions as a critical oncogenic pseudogene in GC by promoting tumor growth through aerobic glycolysis. Mechanistically, ANXA2P1 physically binds to hnRNP F and promotes usage of the proximal polyadenylation site, thereby generating the shorter HK2 3′UTR isoform. Enhanced stability of the short-3′UTR isoform leads to elevated HK2 mRNA abundance and protein expression. In turn, ANXA2P1 expression is transcriptionally induced by the c-Myc/HK2 complex, forming a reinforcing regulatory loop. Therefore, our results identify the ANXA2P1-hnRNP F-HK2/c-Myc feedback loop as a potential therapeutic target for GC.

## Supplementary Material

Supplementary figures.

Supplementary table 1.

Supplementary table 2.

Supplementary table 3.

Supplementary table 4.

Supplementary table 5.

Supplementary table 6.

Supplementary table 7.

Supplementary table 8.

## Data Availability

The microarray, RNA-seq, and RIP-seq data were deposited into the GEO database (accession No. GSE303312, GSE303469, and GSE303348). All other relevant data are available within the article and supplementary files, or from the authors upon reasonable request.

## Abbreviations

2-DG: 2-deoxy-D-glucose
2-NBDG: 2-(N-(7-nitrobenz-2-oxa-1:3-diazol-4-yl)amino)-2-deoxyglucose
3′RACE: 3′ Rapid Amplification of cDNA Ends
3′UTR: 3′ untranslated region
AGPG: Actin Gamma 1 Pseudogene
ANXA2: annexin A2
ANXA2P1: annexin A2 pseudogene 1
APA: Alternative cleavage and polyadenylation
CASC2: cancer susceptibility candidate 2
CDK4/6: cyclin dependent kinase 4/6
CFA: colony formation assays
ChIP: chromatin immunoprecipitation
Co-IP: coimmunoprecipitation
CtBP1/2: C-terminal Binding Protein 1 and 2
DEGs: differentially expressed genes
DSS: disease-specific survival
ECAR: extracellular acidification rate
FC: fold change
G418: geneticin
GC: gastric cancer
GO: gene ontology
H&E: hematoxylin and eosin
HCC: hepatocellular carcinoma
HGNC: HUGO Gene Nomenclature Committee
IHC: immunohistochemistry
IRS: immunoreactive score
LG: low-glucose
LncRNA: long noncoding RNA
MS: mass spectrometry
MT: mutant
NG: normal-glucose
NSCLC: non-small-cell lung cancer
ORF: open reading frame
OS: overall survival
PCAF: p300/CBP-associated factor
PFI: progression-free interval
PI: propidium iodide
Pre-mRNA: precursor mRNA
qRRM: quasi-RNA recognition motif
RAP: RNA antisense purification
RF: random forest
RHAPA: RNase H alternative polyadenylation assay
RIP: RNA-immunoprecipitation
RIP-seq: RNA immunoprecipitation sequencing
RNA-seq: RNA sequencing
SD: standard deviation
SVM: support vector machine
WFDC21P: WAP four-disulfide core domain 21 pseudogene
WT: wild-type
