# Supplementary material for: The ANXA2P1-hnRNP F-HK2/c-Myc Positive Feedback Loop Promotes Proliferation and Glycolytic Metabolism in Gastric Cancer
Source: Int J Biol Sci. 2026 Mar 25;22(7):3658–81. doi: 10.7150/ijbs.126842 (PMC13086010; doi:10.7150/ijbs.126842)
Supplement: Supplementary file 1 — Supplementary figures. [file ijbsv22p3658s1.pdf]

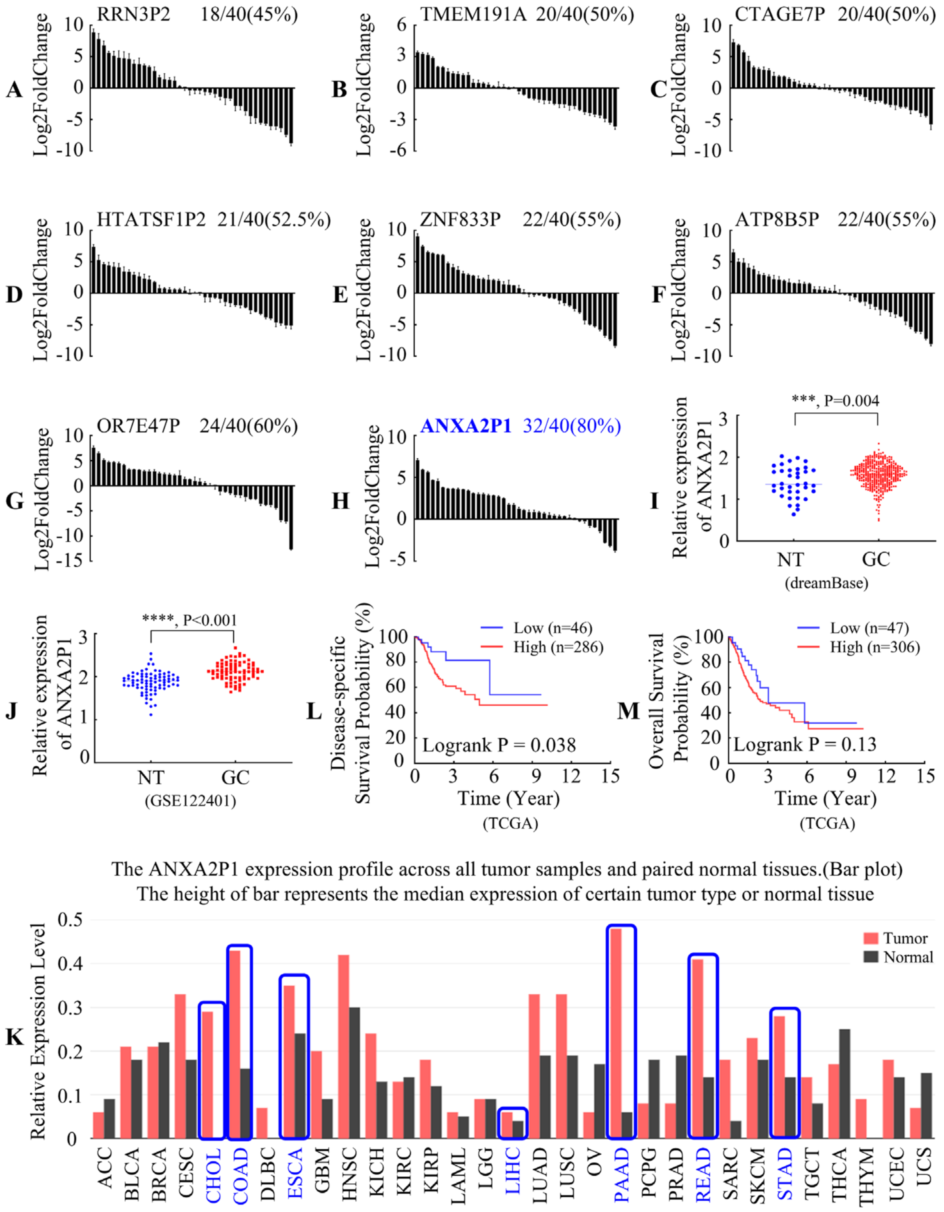

Figure S1. ANXA2P1 is overexpressed and associated with poor prognosis in GC. (A–H) RT-qPCR analysis of eight candidate pseudogenes in GC and adjacent normal tissues (n = 40). **(I–J)** Expression levels of ANXA2P1 from the dreamBase (I) and GSE122401 (J) datasets. **(K)** ANXA2P1 expression pattern in various cancers was generated using the GEPIA database (http://gepia.cancer-pku.cn/). The blue box indicates the expression levels in tumors and normal tissues of the digestive system. **(L–M)** Kaplan–Meier curves for disease-specific survival (L) and overall survival (M) stratified by ANXA2P1 expression levels, based on the TCGA database (source of dreamBase). Mann–Whitney U test (I); Paired Student's t-test (J); Kaplan–Meier survival analysis with log-rank test (L–M). ***$P < 0.01$ and ****$P < 0.001$.

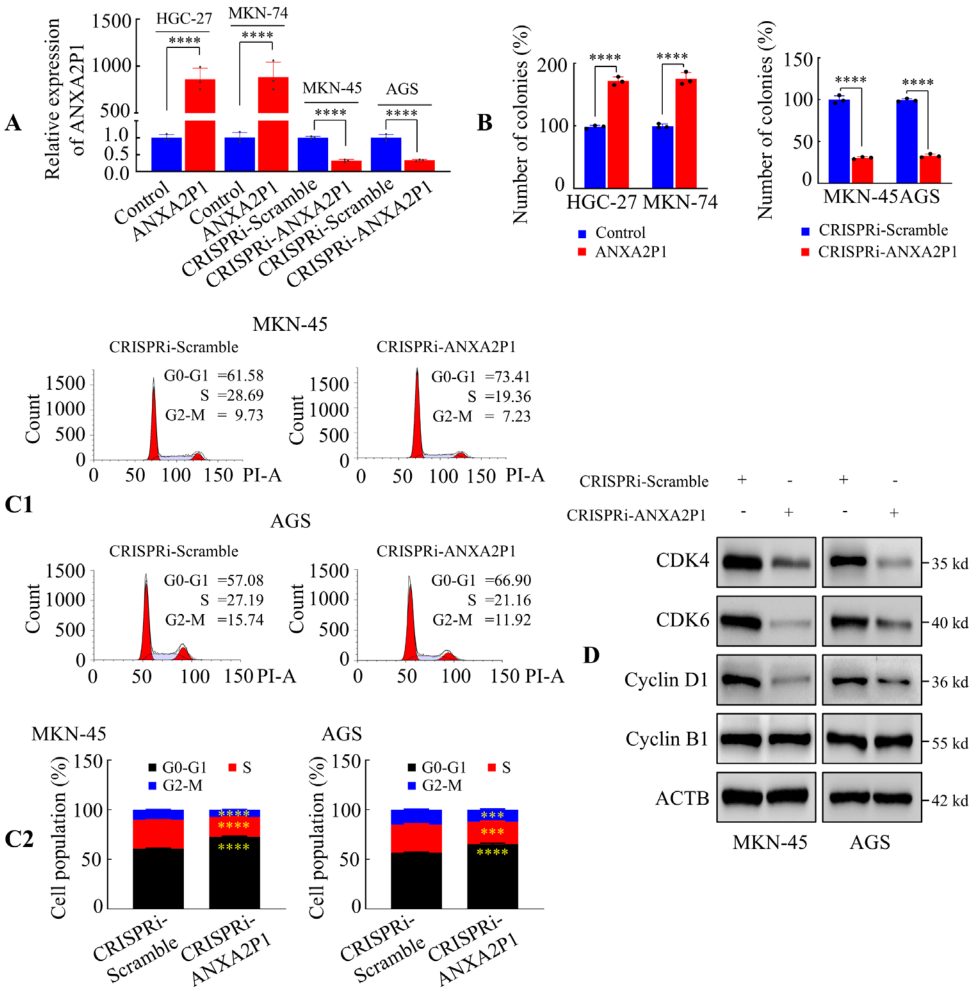

Figure S2. The biological function analysis of ANXA2P1 in GC cells. **(A)** RT-qPCR analysis of ANXA2P1 knockdown efficiency in the indicated *GC* cell lines. **(B)** Quantification of crystal violet-stained colonies formed by the indicated GC cell lines. **(C1/2)** Flow cytometry analysis of cell cycle distribution in GC cells stained with propidium iodide (PI) (C1). The distribution and percentage of cells in G1, S, and G2/M phases are displayed (C2). CRISPRi-Scramble versus CRISPRi-ANXA2P1. **(D)** Western blotting of cell cycle-related proteins in GC cells. Student's t-test (A–C). ****P* < 0.01 and *****P* < 0.001. Data are presented as mean ± SD.

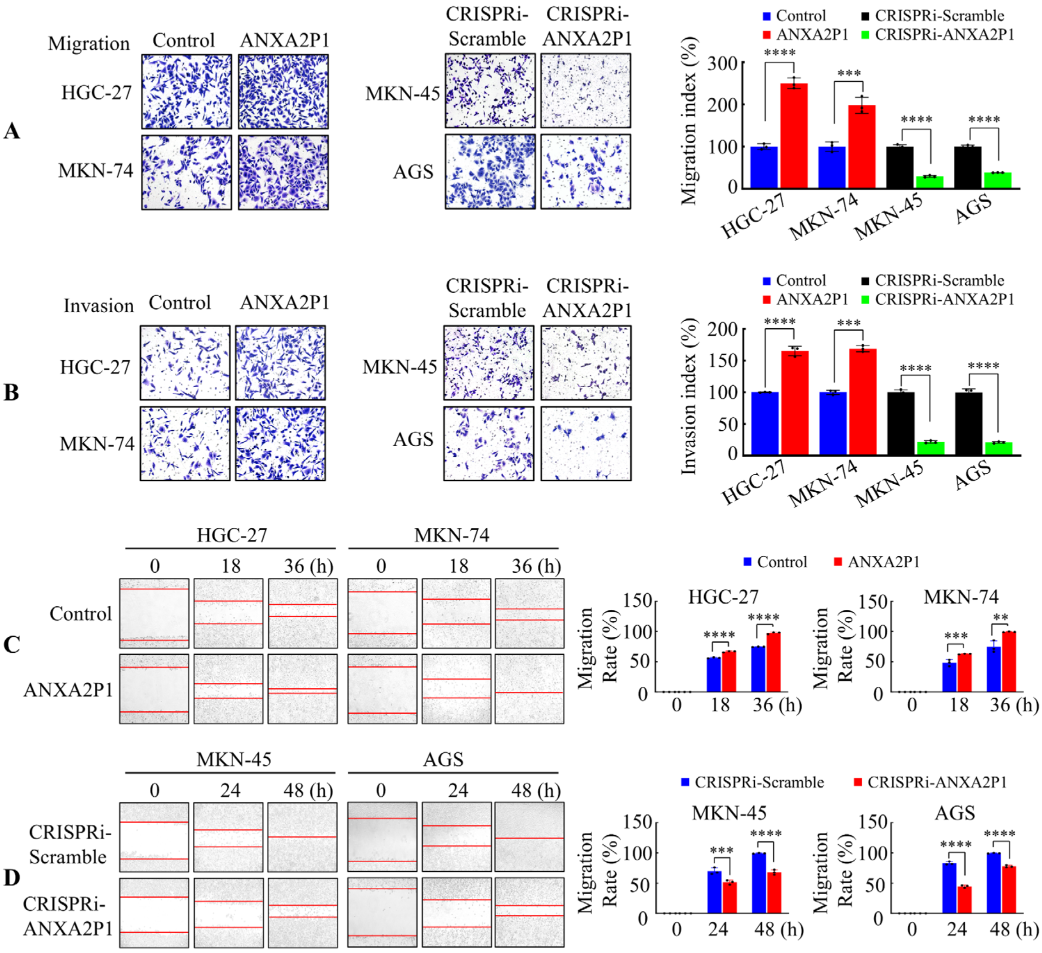

Figure S3. The biological function analysis of ANXA2P1 in GC cells. **(A–B)** Migration (A) and invasion (B) assays were performed using the indicated GC cells. **(C–D)** The wound healing assay was performed to assess GC cell motility. Student's t-test (A–D). $^{**}P < 0.05$, $^{***}P < 0.01$, and $^{****}P < 0.001$. Data are presented as mean ± SD.

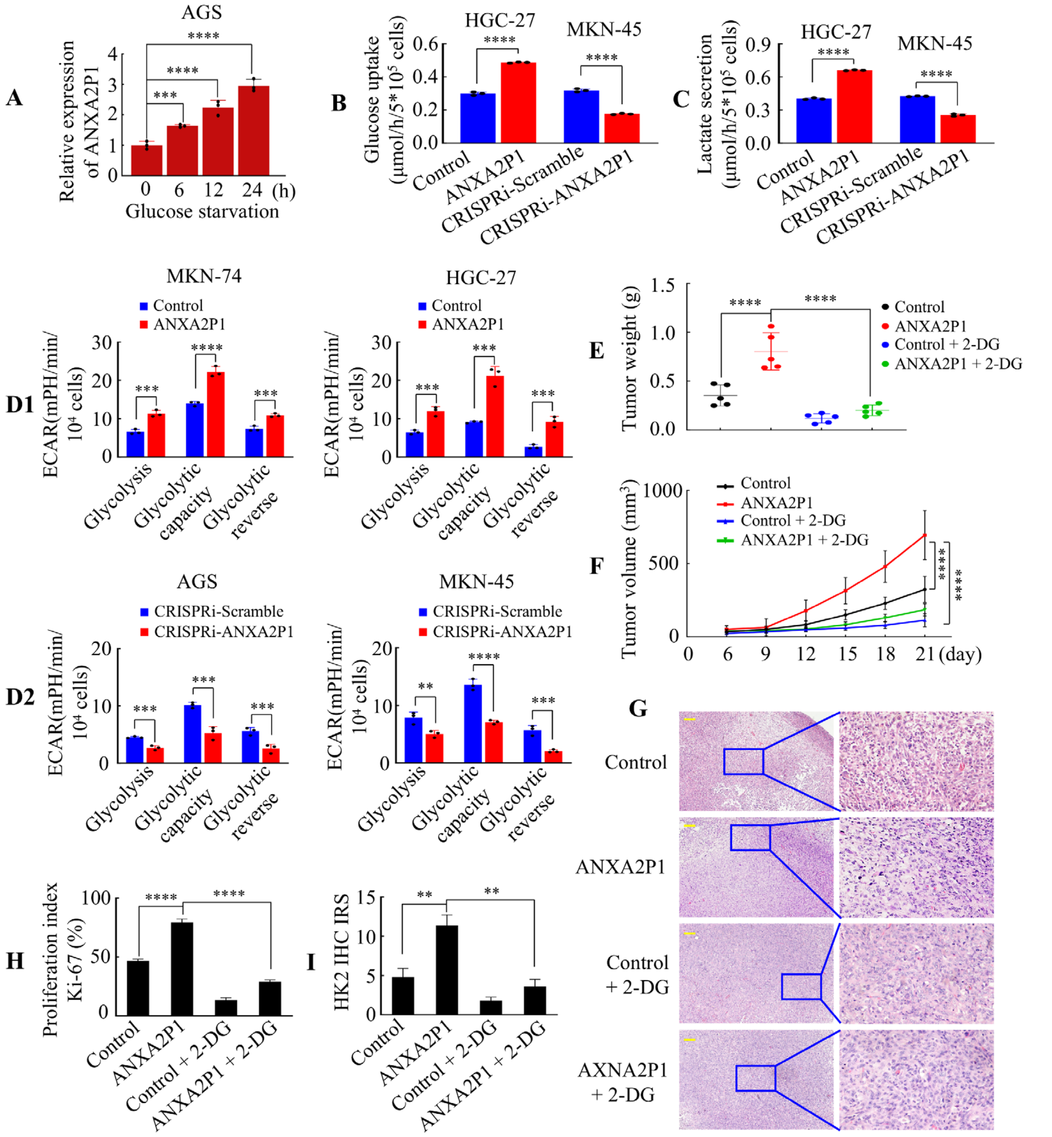

Figure S4. ANXA2P1 promotes the glycolysis of GC cells in vitro and in vivo. (A) RT-qPCR analysis of ANXA2P1 expression in AGS cells after incubation in low-glucose medium for 0, 6, 12, and 24 h. **(B–C)** Glucose consumption (B) and lactate level (C) were detected in the supernatants of HGC-27 and MKN-45 cells. **(D1/2)** Statistical analysis of the effects of ANXA2P1 on ECAR. **(E)** Tumor weights were assessed after the indicated treatments (n = 5). **(F)** The tumor volumes were measured in mice following application of different treatments (n = 5). Control versus ANXA2P1 and ANXA2P1 versus ANXA2P1 + 2-DG. **(G)** Representative images of H&E staining of subcutaneous tumor samples. **(H–I)** Quantification of Ki-67 (H) and HK2 (I) expression by IHC in subcutaneous tumors from mice injected with MKN-74 cells. IRS, Immunoreactive Score. Scale bar: 100 μm in (G). One-way ANOVA, Dunnett's multiple comparisons test (A, E, F, and H); Student's t-test (B–D); Kruskal–Wallis test, Dunn's multiple comparisons test (I). $^{**}P < 0.05$, $^{***}P < 0.01$, and $^{****}P < 0.001$. Data are presented as mean ± SD.

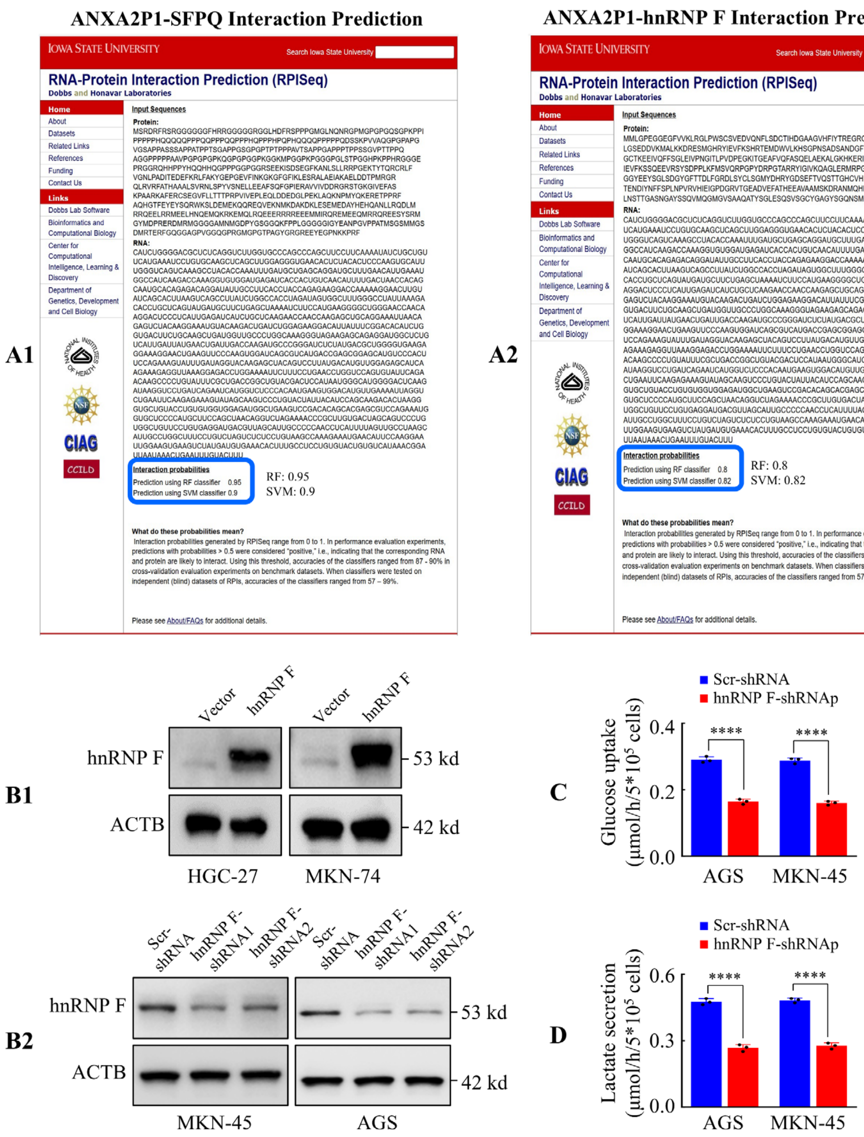

Figure S5. ANXA2P1 potentially interacts with SFPQ or hnRNP F. (A1/2) RNA-protein interactions of ANXA2P1-SFPQ (A1) and ANXA2P1-hnRNP F (A2) were predicted using the RPISeq database with Random Forest and Support Vector Machine models. Interactions with a probability score above 0.50 were considered positive. **(B1/2)** Verification of hnRNP F overexpression (B1) and knockdown (B2) efficiency in GC cells by Western blotting. **(C–D)** Glucose consumption (C) or lactate level (D) was measured in the supernatants of AGS and MKN-45 cells after transfection with hnRNP F-shRNAp or Scr-shRNA. Student's t-test (C–D). ****$P < 0.001$. Data are presented as mean ± SD.

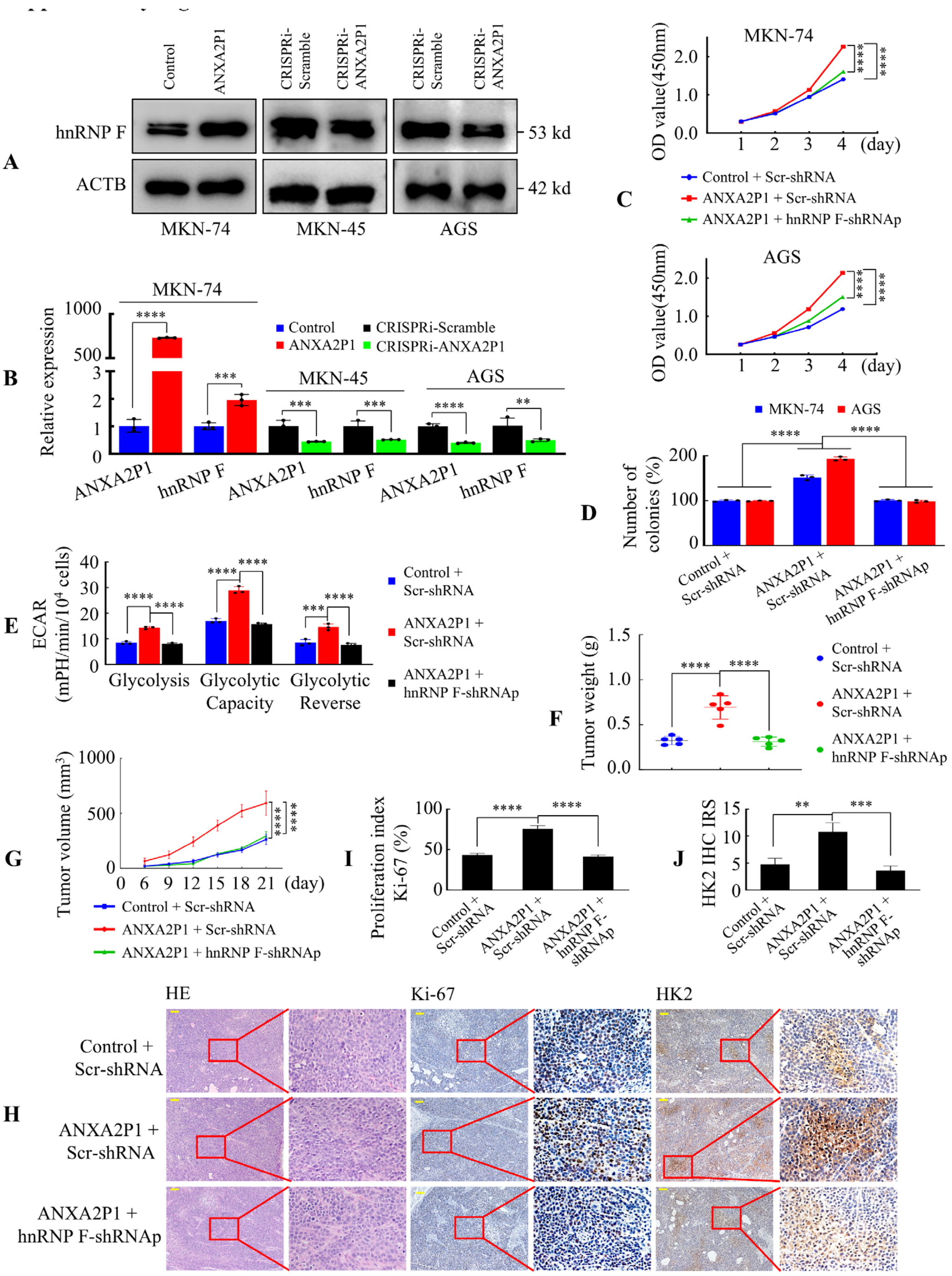

Figure S6. ANXA2P1 promotes cell proliferation and glycolysis via hnRNP F. (A–B) Western blotting (A) and RT-qPCR (B) analyses of hnRNP F expression levels after ANXA2P1 overexpression and silencing. **(C–D)** CCK-8 (C) and quantification of CFA (D) in the indicated GC cells. Control + Scr-shRNA versus ANXA2P1 + Scr-shRNA, and ANXA2P1 + Scr-shRNA versus ANXA2P1 + hnRNP F-shRNAp. **(E)** Statistical analysis of ECAR across the indicated groups. **(F)** Tumor weights were assessed in mice after the indicated treatments (n = 5). **(G)** Tumor volumes measured in each group. Control + Scr-shRNA versus ANXA2P1 + Scr-shRNA, and ANXA2P1 + Scr-shRNA versus ANXA2P1 + hnRNP F-shRNAp. **(H)** Representative images of H&E staining and IHC staining for Ki-67 and HK2. **(I–J)** Quantification of Ki-67 (I) and HK2 (J)

expression by IHC in subcutaneous tumors. Scale bar: 100 μm in (H). Student's t-test (B); one-way ANOVA, Dunnett's multiple comparisons test (C–I); Kruskal–Wallis test, Dunn's multiple comparisons test (J). **$P < 0.05$, ***$P < 0.01$, and ****$P < 0.001$. Data are presented as mean ± SD.

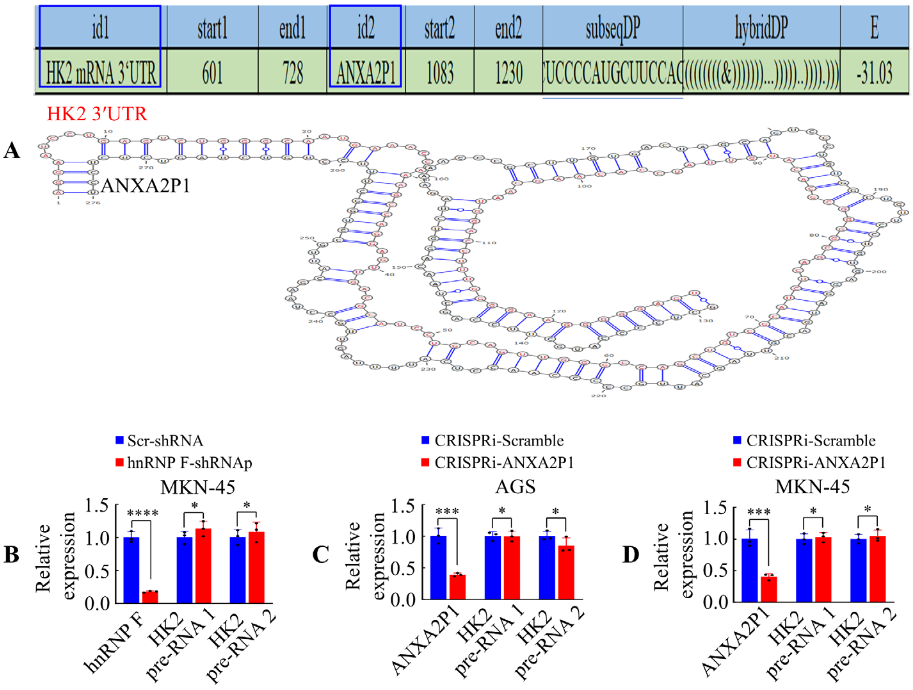

Figure S7. ANXA2P1 and hnRNP F interact with HK2 3′UTR to modulate HK2 expression at post-transcriptional level. (A) IntaRNA was used to search for complementary regions between ANXA2P1 and the HK2 3′UTR, with a predicted binding free energy of ΔG = –31.03 kcal/mol. **(B–D)** RT-qPCR analysis of HK2 pre-mRNA levels in GC cells following indicated treatment. Student's t-test. *P > 0.05, ***P < 0.01, and ****P < 0.001. Data are presented as mean ± SD.

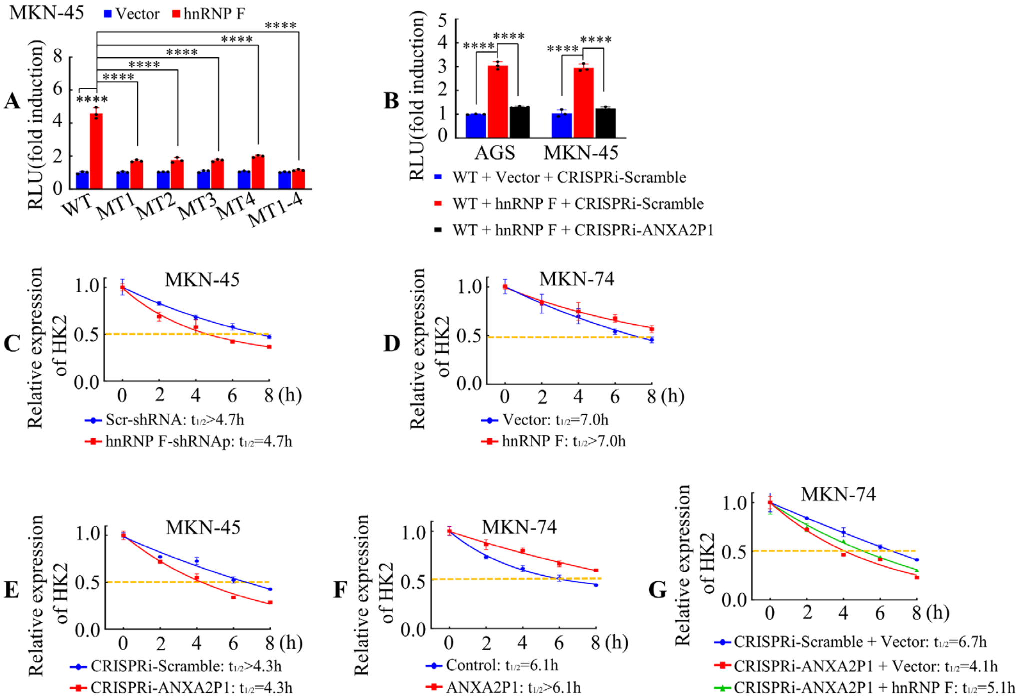

Figure S8. ANXA2P1 and hnRNP F collaboratively regulate HK2 mRNA stability. **(A)** Relative luciferase activities of HK2 3′UTR in MKN-45 cells after the displayed treatment. WT, Wild type; MT, Mutation. **(B)** Relative luciferase activity of the HK2 3′UTR after indicated transfection in AGS and MKN-45 cells. **(C–F)** Assessment of HK2 mRNA half-life following modulation of hnRNP F (C–D) or ANXA2P1 (E–F) in GC cells. **(G)** Assessment of the cooperative effects of ANXA2P1 and hnRNP F on HK2 mRNA half-life. One-way ANOVA, Dunnett's multiple comparisons test (A–B). ****$P < 0.001$. Data are presented as mean ± SD.

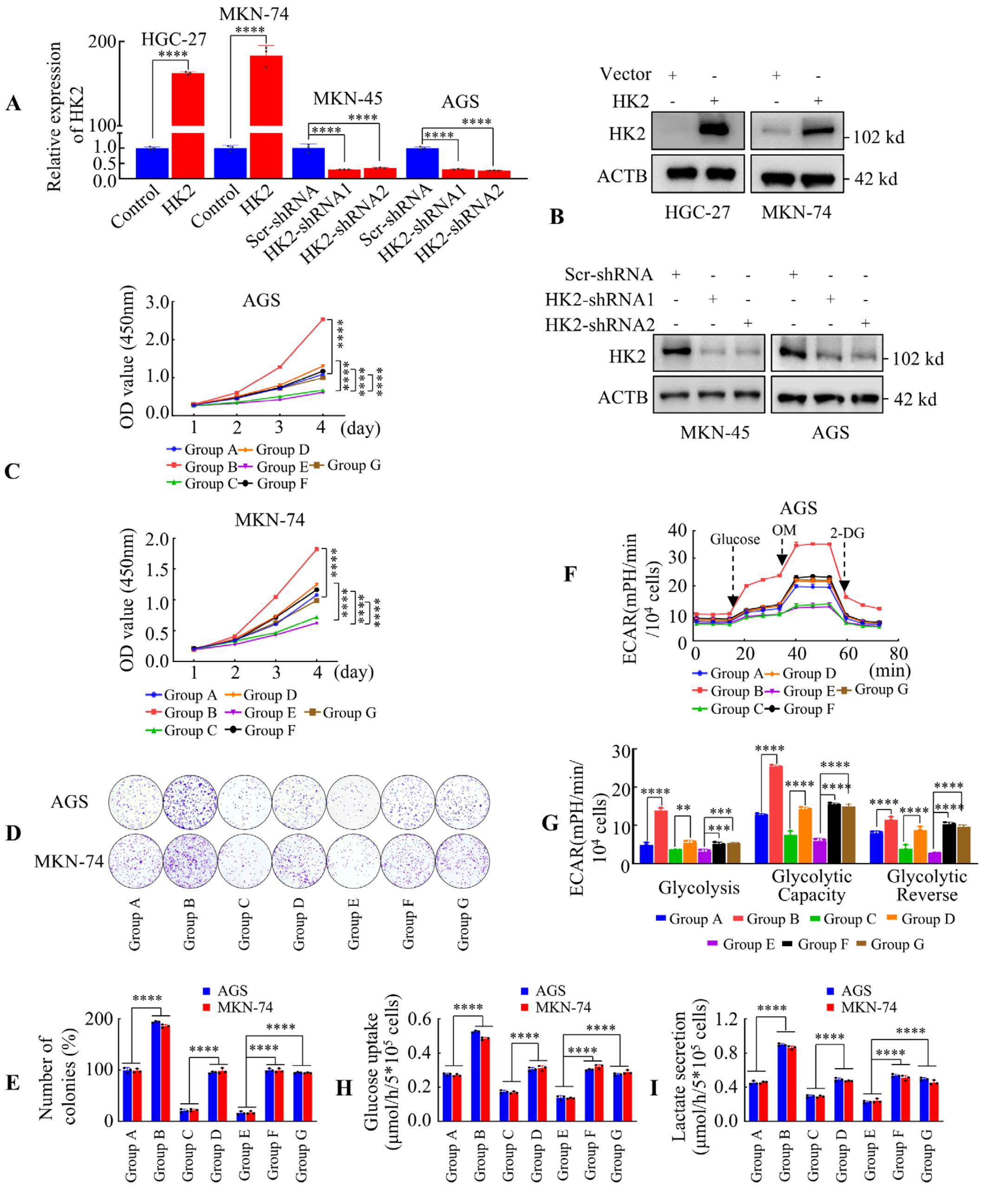

Figure S9. Co-regulation of ANXA2P1 and hnRNP F modulates HK2 expression to promote GC cell proliferation and glycolysis. (A–B) Verification of HK2 overexpression or knockdown efficiency in GC cells by RT-qPCR (A) and Western blotting (B) assays. **(C–I)** Group designations: Group A, CRISPRi-Scramble + Scr-shRNA + Vector; Group B, CRISPRi-Scramble + Scr-shRNA + HK2; Group C, CRISPRi-ANXA2P1 + Scr-shRNA + Vector; Group D, CRISPRi-ANXA2P1 + Scr-shRNA + HK2; Group E, CRISPRi-Scramble + hnRNP F-shRNAp + Vector; Group F, CRISPRi-Scramble + hnRNP F-shRNAp + HK2; and Group G, CRISPRi-ANXA2P1 + hnRNP F-shRNAp + HK2. **(C)** CCK-8 assay was performed in the indicated cells. Group A versus Group B, Group C versus Group D, and Group E versus Group F or Group G. **(D–E)** Colony formation assays and corresponding statistical analysis in the

indicated GC cells. **(F–G)** ECAR and corresponding statistical analysis in the displayed AGS cells. **(H–I)** Glucose consumption (H) or lactate level (I) was measured in the supernatants of MKN-74 and AGS cells. Student's t-test (A); **o**ne-way ANOVA, Tukey's multiple comparisons test (A–I). $**P < 0.05$, $***P < 0.01$, and $****P < 0.001$. Data are presented as mean ± SD.

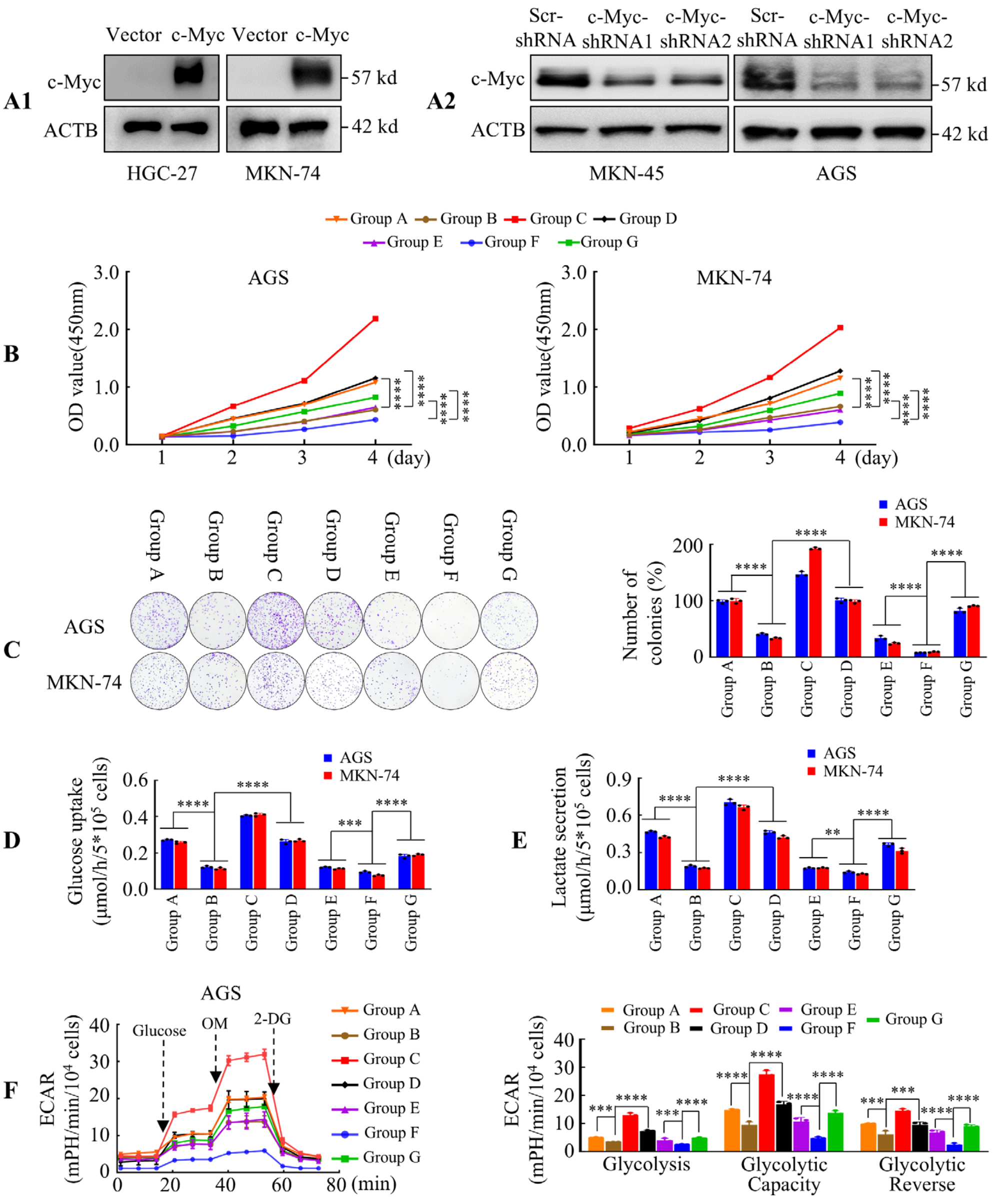

Figure S10. ANXA2P1 is modulated by c-Myc and HK2 to promote GC proliferation and glycolysis in vitro. (A1/2) Validation of c-Myc overexpression (A1) or knockdown (A2) efficiency by Western blotting in GC cells. **(B–F)** Group designations: Group A, Scr-shRNA + control; Group B, c-Myc-shRNAp + control; Group C, Scr-shRNA + ANXA2P1; Group D, c-Myc-shRNAp + ANXA2P1; Group E, c-Myc-shRNAp + Scr-shRNA + control; Group F, c-Myc-shRNAp + HK2-shRNAp + control; Group G, c-Myc-shRNAp + HK2-shRNAp + ANXA2P1. **(B)** CCK-8 assay was performed in GC cells transfected as illustrated in the figure. Group B versus Group A or Group D, Group F versus Group E or Group G. **(C)** CFA assay and corresponding quantification in GC cells as illustrated. **(D–E)** Glucose consumption (D) and lactate production (E) were detected with the indicated treatment. **(F)** ECAR and corresponding statistical analysis in GC cells with the indicated treatment. One-way ANOVA, Dunnett's multiple comparisons test (B–F). **P < 0.05, ***P < 0.01, and ****P < 0.001. Data are presented as mean ± SD.

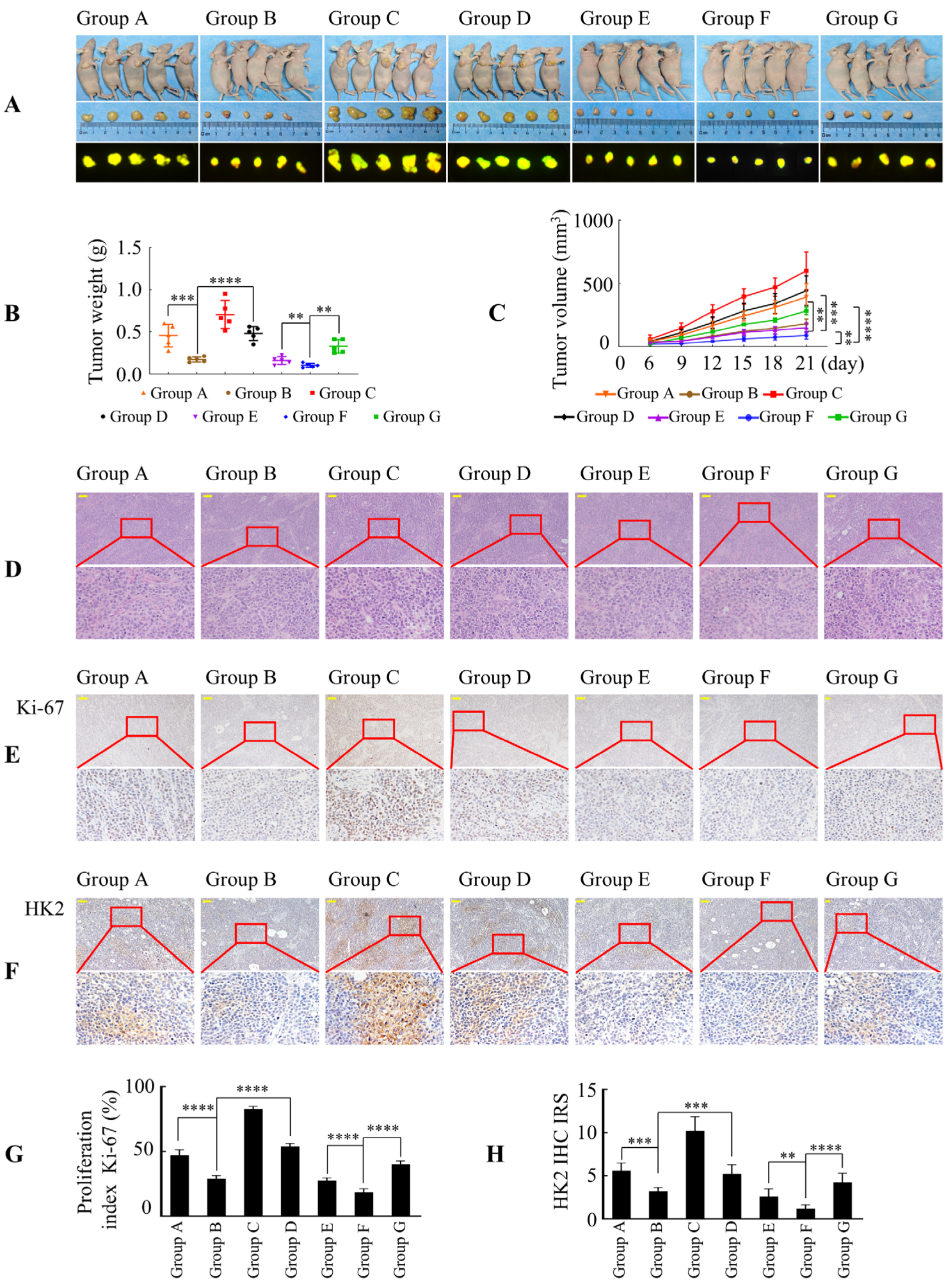

Figure S11. ANXA2P1 is modulated by c-Myc and HK2 to promote GC proliferation and glycolysis in vivo. Group designations: Group A, Scr-shRNA + control; Group B, c-Myc-shRNAp + control; Group C, Scr-shRNA + ANXA2P1; Group D, c-Myc-shRNAp + ANXA2P1; Group E, c-Myc-shRNAp + Scr-shRNA + control; Group F, c-Myc-shRNAp + HK2-shRNAp + control; Group G, c-Myc-shRNAp + HK2-shRNAp + ANXA2P1. **(A)** Representative images of subcutaneous tumors in mice (n = 5). **(B)** Tumor weights were assessed in mice after the indicated treatments (n = 5). **(C)** Tumor volumes were measured in each group (n = 5). **(D)** Representative images of H&E staining of subcutaneous tumor samples in the indicated groups. **(E–H)** Representative IHC staining images and corresponding quantification of Ki-67 (E and G) or HK2 (F and H). IRS, Immunoreactive Score.

Scale bar: 100 µm. One-way ANOVA, Dunnett's multiple comparisons test (B, C, and G); Kruskal–Wallis test, Dunn's multiple comparisons test (H). **P < 0.05, ***P < 0.01, and ****P < 0.001. Data are presented as mean ± SD.

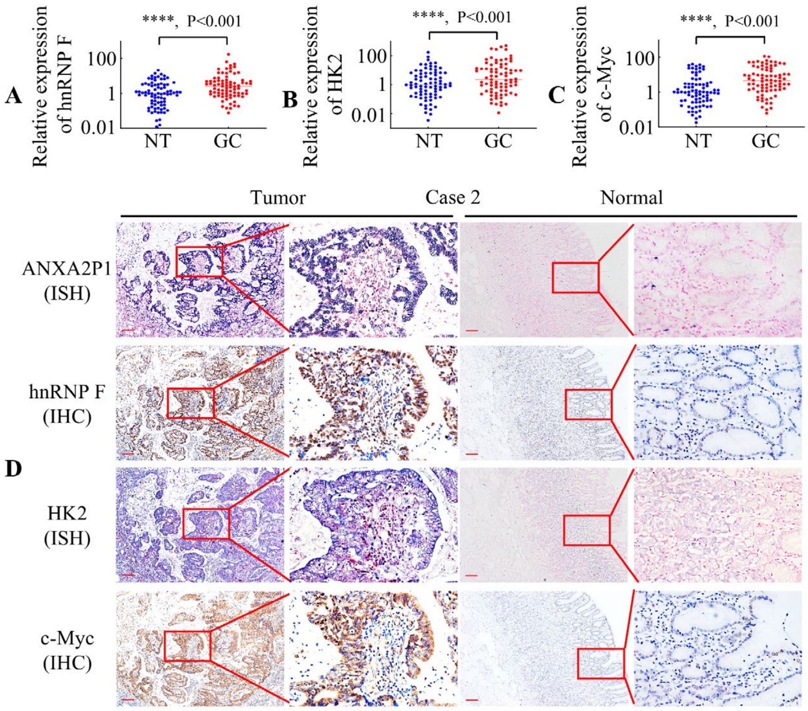

Figure S12. ANXA2P1 expression levels are positively correlated with hnRNP F, HK2, and c-Myc expression in GC tissues. (A–C) Scatter plots illustrating expression levels of hnRNP F (A), HK2 (B), and c-Myc (C) in 80 paired GC and adjacent normal tissues, as measured by RT-qPCR. **(D)** Representative ISH and IHC images display the expression of the indicated genes in GC and adjacent normal tissues. Scale bar: 100 μm. Paired two-sided Student's t-test (A–C). ****P < 0.001.
