# Supplementary material for: The ANXA2P1-hnRNP F-HK2/c-Myc Positive Feedback Loop Promotes Proliferation and Glycolytic Metabolism in Gastric Cancer
Source: Int J Biol Sci. 2026 Mar 25;22(7):3658–81. doi: 10.7150/ijbs.126842 (PMC13086010; doi:10.7150/ijbs.126842)
Supplement: Supplementary file 3 — Supplementary table 2. [file ijbsv22p3658s3.pdf]

Supplementary Table 2A. Correlation between ANXA2P1 expression and clinicopathological parameters of GC

**Parameters**	**Case**	**ANXA2P1 expression**	**p-value**
**Gender**			
Female	24	2.350(1.385,12.847)	0.652
Male	56	3.313(1.121,11.962)	
**Age (years)**			
<60	37	3.349(0.807,12.655)	0.579
≥60	43	2.336(1.432,12.498)	
**Tumor size (cm)**			
<5	47	1.761(1.093,7.047)	0.001
≥5	33	8.486(2.139,29.885)	
**Histological grade**			
Well/Moderate	25	1.933(0.814,5.255)	0.042
Poor/Undifferentiated	55	5.633(1.334,13.899)	
**Local invasion (pT status)**			
T1/T2	17	1.761(0.675,3.660)	0.034
T3/T4	63	4.331(1.334,13.899)	
**Lymph node (pN status)**			
Absent (N0)	21	1.653(0.809,2.290)	0.004
Present (N1-3)	59	7.047(1.373,13.899)	
**Distant metastasis (pM status)**			
M0	73	2.209(1.168,11.267)	<0.001
M1	7	60.335(12.498,80.851)	
**AJCC stage**			
I/II	28	1.639(0.849,2.330)	0.001
III/IV	52	7.149(1.517,22.257)	

Supplementary Table 2B. Correlation between hnRNP F expression and clinicopathological parameters of GC

**Parameters**	**Case**	**hnRNP F expression**	**p-value**
**Gender**			
Female	24	2.774(0.802,6.861)	0.622
Male	56	2.998(0.873,14.352)	
**Age (years)**			
<60	37	3.421(1.018,12.524)	0.612
≥60	43	2.643(0.673,14.531)	
**Tumor size (cm)**			
<5	47	1.616(0.513,4.151)	0.001
≥5	33	6.208(2.481,24.871)	
**Histological grade**			
Well/Moderate	25	1.747(0.80,9.441)	0.369
Poor/Undifferentiated	55	3.421(0.844,12.750)	
**Local invasion (pT status)**			
T1/T2	17	0.844(0.437,5.924)	0.079
T3/T4	63	3.075(1.194,13.802)	
**Lymph node (pN status)**			
Absent (N0)	21	0.615(0.200,2.334)	<0.001
Present (N1-3)	59	3.821(1.594,14.531)	
**Distant metastasis (pM status)**			
M0	73	2.645(0.657,7.903)	0.001
M1	7	27.606(9.181,33.90)	
**AJCC stage**			
I/II	28	0.989(0.467,3.678)	0.003
III/IV	52	3.797(1.637,14.352)	

Supplementary Table 2C. Correlation between HK2 expression and clinicopathological parameters of GC

**Parameters**	**Case**	**HK2 expression**	**p-value**
**Gender**			
Female	24	2.645(0.465,9.610)	0.319
Male	56	4.037(1.139,15.637)	
**Age (years)**			
<60	37	3.783(0.929,13.180)	0.973
≥60	43	3.710(0.402,12.031)	
**Tumor size (cm)**			
<5	47	1.723(0.355,6.231)	0.001
≥5	33	5.306(3.349,17.006)	
**Histological grade**			
Well/Moderate	25	1.791(0.455,5.769)	0.053
Poor/Undifferentiated	55	5.018(1.138,15.754)	
**Local invasion (pT status)**			
T1/T2	17	1.613(0.181,2.128)	0.009
T3/T4	63	4.823(1.330,15.680)	
**Lymph node (pN status)**			
Absent (N0)	21	1.141(0.156,2.507)	<0.001
Present (N1-3)	59	5.082(1.613,17.517)	
**Distant metastasis (pM status)**			
M0	73	2.549(0.522,9.271)	0.027
M1	7	12.031(4.823,27.755)	
**AJCC stage**			
I/II	28	1.650(0.196,3.420)	<0.001
III/IV	52	5.177(1.820,17.058)	

Supplementary Table 2D. Correlation between c-Myc expression and clinicopathological parameters of GC

Parameters	Case	c-Myc expression	p-value
**Gender**			
Female	24	6.779(1.364,10.772)	0.817
Male	56	4.022(1.265,15.035)	
**Age (years)**			
<60	37	6.543(1.480,14.488)	0.534
≥60	43	3.348(1.206,12.682)	
**Tumor size (cm)**			
<5	47	3.000(1.009,9.331)	0.005
≥5	33	9.682(1.763,41.381)	
**Histological grade**			
Well/Moderate	25	1.492(0.766,5.130)	0.008
Poor/Undifferentiated	55	7.862(2.256,14.563)	
**Local invasion (pT status)**			
T1/T2	17	1.492(0.677,9.584)	0.024
T3/T4	63	5.230(1.572,15.209)	
**Lymph node (pN status)**			
Absent (N0)	21	1.141(0.223,4.410)	<0.001
Present (N1-3)	59	7.862(2.057,17.586)	
**Distant metastasis (pM status)**			
M0	73	3.348(1.190,13.386)	0.011
M1	7	12.444(9.331,32.139)	
**AJCC stage**			
I/II	28	1.719(0.653,5.541)	0.001
III/IV	52	7.878(2.064,21.291)	
