# Supplementary material for: The ANXA2P1-hnRNP F-HK2/c-Myc Positive Feedback Loop Promotes Proliferation and Glycolytic Metabolism in Gastric Cancer
Source: Int J Biol Sci. 2026 Mar 25;22(7):3658–81. doi: 10.7150/ijbs.126842 (PMC13086010; doi:10.7150/ijbs.126842)
Supplement: Supplementary file 9 — Supplementary table 8. [file ijbsv22p3658s9.pdf]

Supplementary Table S8A. List of primers

Experiment	Name	Position or orientation	Sequence (5’-3’)
RT-qPCR	ANXA2P1	F	CCAGCAAGACACTAAGGGTGC
		R	CCAGGCAATGCTTAGGCAAC
	ATP8B5P	F	CAGTTGGGCATGGCATTTGG
		R	TGGGCACCATGGTATTGAGA
	CTAGE7P	F	TTCCGCCTTCAAATGAGCCT
		R	GCAGTTACTTGAAATGAGATCAGTC
	HTATSF1P2	F	ATGCTAAAGCCACAGGGGAA
		R	TTGCAACTGCCCCAAGTTCT
	OR7E47P	F	GTTCCCGAAGAAGCTCGGT
		R	GCCCCAGCAGTGTTCTTCT
	TMEM191A	F	ACTTGAAGCCCGGTAAACCC
		R	TGCAGTTCCCCTCCGTAGTA
	ZNF833P	F	AGAGATCTTCAGCCTTGTTCCAA
		R	TGTCAGCTCTGATGCAGCAAT
	RRN3P2	F	CGCAGCTTAGTGGCTCAGTT
		R	TGTATGGGCCGGCTTTATGT
	SUMO1P3	F	TACGTGTGCACAGAGAGCC
		R	CCAATTCGCAAGTTATTATGAACG
	ACTB	F	CATGTACGTTGCTATCCAGGC
		R	CTCCTTAATGTCACGCACGAT
	U6	F	CTCGCTTCGGCAGCACA
		R	AACGCTTCACGAATTTGCGT
	B2M	F	ACTGAATTCACCCCCACTGA
		R	CCTCCATGATGCTGCTTACA
	HNRNPF	F	ACATTTACAACTTCTTCTCTC
		R	AACTCAACATCTGCTTCA
	HK2	F	GAGCCACCACTCACCCTACT
		R	CCAGGCATTCGGCAATGTG
	HK2 pre-mRNA-1	F	CAGATGGGACAGGTACTGCAT
		R	GCCACAGTAGGGCTGACAAA
	HK2 pre-mRNA-2	F	GATGGGACAGGTACTGCATC
		R	CCTGTCCATCCGTCACACAG
	HK2 common 3′UTR (shared with short isoform)	F	CCCCTGAAATCGGAAGGGAC
		R	CAAGCAAAAGCCAAGGGGTC
	Long HK2 3′UTR	F	GTGTAGCTCCTCTGCTGCTC
		R	TTTGTGGGCACCCTTTAGTGA

	MYC	F	CCCTCCACTCGGAAGGACTA
		R	GCTGGTGCATTTTCGGTTGT
	IRF7	F	AGCCCTTACCTCCCCTGTTA
		R	GCCCTCTCAGGAGCCAAG
ChIP and re-ChIP (ANXA2P1 promoter)	Site1	F: +96 ~ +117	ACACTCTACACTCCCAAGTGC
		R: +234 ~ +254	GTCTCTGTGCATTGCTGTGG
	Site2	F: -506 ~ -485	CGAGATCATCAGAGACAGGGG
		R: -425 ~ -404	ACCAAAACCCAGAAGAAGCCA
DNA pull-down probe (ANXA2P1 promoter)		F	AGGTGCCAGAACATTTCTCTATCG ATAGGTACCCTTTCATAACAAATA TAAATAACACAAAACACATATA
		R	TGGCTTTACCAACAGTACCGGAA TGCCAAGCTTTGTACATTTCCTTG TAGACTCTGTTAATTTCCTGCA
3′RACE	Gene-specific primer	Outer	TTAAGGAGGTGTGCACTGTGG
		Inner	GAGAAAACCGTGGGCTGGA
RHAPA	DNA oligonucleotide		CCAAGGTGAAGCAACCGTAT
	RNase H cleavage confirmation	F	GGCTGAGCTTGGCCCTATT
		R	CAACATCAGTGCTGGGGGAT

Supplementary Table S8B. List of antibodies used in WB, CoIP, ChIP, RIP, and IHC

Antibody	Vendor	Catalog No.	Application	
CDK4	Proteintech	11026-1-AP	WB	1:4000
CDK6	Proteintech	66278-1-Ig	WB	1:5000
Cyclin D1	CST	2978S	WB	1:1000
Cyclin B1	Proteintech	28603-1-AP	WB	1:8000
ACTB	Proteintech	66009-1-Ig	WB	1:25000
Ki-67	Proteintech	27309-1-AP	IHC	1:5000
HK2	Proteintech	22029-1-AP	WB	1:20000
			IHC	1:200
hnRNP F	Proteintech	14974-1-AP	IHC	1:50
	Santa Cruz	32309	WB	1:200
			RIP	5μg

SFPQ	ABclonal	A0958	WB	1:500
			RIP	5μg
c-Myc	CST	5605	WB	1:1000
	Proteintech	10828-1-AP	ChIP	2μg
			CoIP	2μg
			IHC	1:100
Flag	Abmart	M20008S	WB	1:20000
			RIP	5μg
His	Proteintech	66005-1-Ig	ChIP	2μg
			WB	1:5000
DIG	Sigma-Aldrich	11093274910	ISH	1:100
IgG-RB	Millipore	12-370	RIP	5μg
			ChIP	2μg
			CoIP	2μg
IgG-MS	Sigma-Aldrich	12-371	RIP	5μg
			ChIP	2μg
			CoIP	2μg

Supplementary Table S8C. The sequences of sgRNAs/shRNAs

Name	sgRNA/shRNA	Sense (5'-3')
ANXA2P1	sgRNA1	TATTTTCAGCTCAGCATCTG
	sgRNA2	CAGATATTTTGAAGGAAGCT
hnRNP F	shRNA1	CCCTGTGAGAGTCCATATT
	shRNA2	GGATGCACAAAGGAAGAAA
HK2	shRNA1	CCAGAAGACATTAGAGCAT
	shRNA2	GCAGAAGGTTGACCAGTAT
c-Myc	shRNA1	GGCGAACACACAACGTCTT
	shRNA2	GCTTGTACCTGCAGGATCTTT

IRF7	shRNA1	GATCAGCAGCGGCTGCTATTT
	shRNA2	CCAACAGCCTCTATGACGATT
Scramble	sgRNA	GCACTCACATCGCTACATCA
	shRNA	TTCTCCGAACGTGTCACGT
